# Supplementary material for: A path towards high lithium-metal electrode coulombic efficiency based on electrolyte interaction motif descriptor
Source: Nat Commun. 2025 May 20;16:4672. doi: 10.1038/s41467-025-59955-0 (PMC12092653; doi:10.1038/s41467-025-59955-0)
Supplement: Supplementary file 1 — Supplementary Information [file 41467_2025_59955_MOESM1_ESM.pdf]

Supplementary Information

A path towards high lithium-metal electrode coulombic efficiency based on electrolyte interaction motif descriptor

Ruhong Li[1, 2, †], Xiaoteng Huang[1, †], Haikuo Zhang[1], Jinze Wang[1], Yingzhu Fan[3], Yiqiang Huang[1], Jia Liu[4], Ming Yang[5], Yuan Yu[6], Xuezhang Xiao[1], Yuanzhong Tan[7], Hao Bin Wu[1], Liwu Fan[4], Tao Deng[8], Lixin Chen[1], Yanbin Shen[3], and Xiulin Fan[1, *]

1. State Key Laboratory of Silicon and Advanced Semiconductor Materials, School of Materials Science and Engineering, Zhejiang University, Hangzhou 310027, China.
2. ZJU-Hangzhou Global Scientific and Technological Innovation Center, Zhejiang University, Hangzhou 311215, China
3. i-Lab, CAS Center for Excellence in Nanoscience, Suzhou Institute of Nano-Tech and Nano-Bionics (SINANO), Chinese Academy of Sciences, Suzhou 215123, P. R. China.
4. State Key Laboratory of Clean Energy Utilization, School of Energy Engineering, Zhejiang University, Hangzhou, 310027, China.
5. Science and Technology on Power Sources Laboratory, Tianjin Institute of Power Sources, Tianjin 300384, China.
6. Zhejiang Provincial Key Laboratory of Fiber Materials and Manufacturing Technology, Zhejiang Sci-Tech University, Hangzhou 310018, China
7. Zhejiang Xinan Chemical Industrial Group Co. Ltd, Hangzhou 311600, P. R. China.
8. China-UK Low Carbon College, Shanghai Jiao Tong University, Shanghai 201306, China.

† These authors contributed equally to this work.

* Corresponding author: xlfan@zju.edu.cn.

Supplementary Figures……………………………………………………1-47

Supplementary Tables……………………………………………………48-59

Supplementary references……………………………………………………60

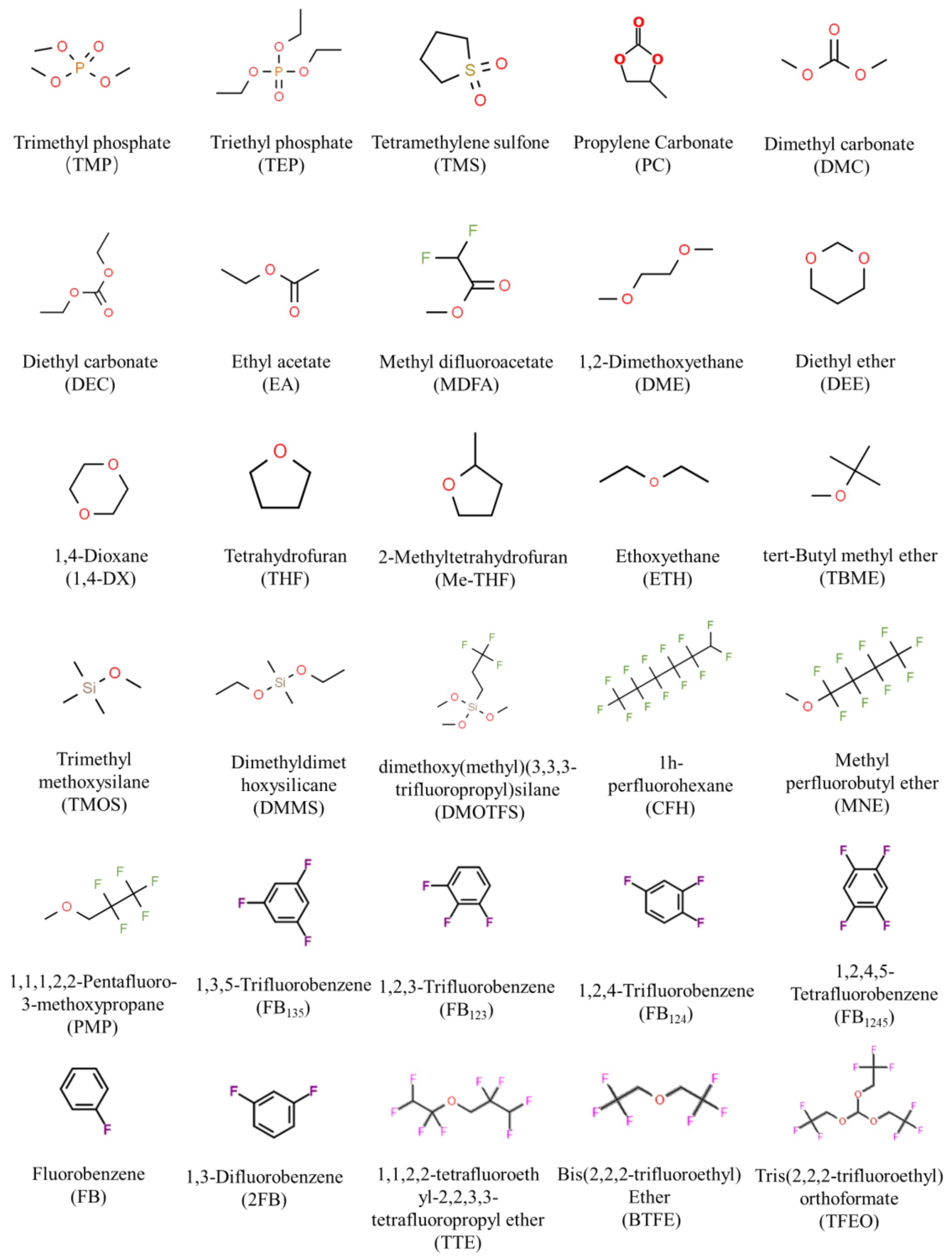

Supplementary Fig. 1 Molecular structures of solvents studied in this work.

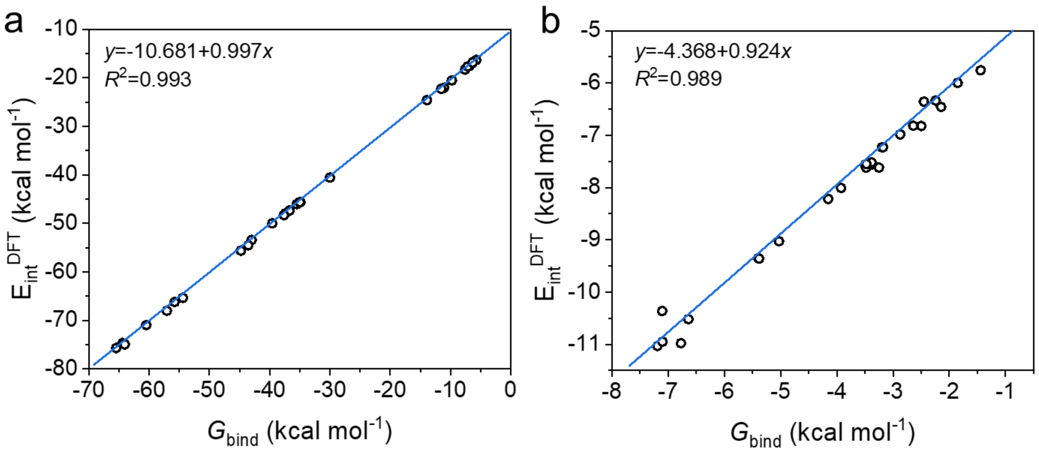

Supplementary Fig. 2 Comparison between the interaction energy of studied dimer complexes computed at the DFT and SAPT method. a Li^+-solvent interactions, **b**, FSI^--solvent interactions. the linear correlation coefficient R^2 between the interaction energies evaluated at the two different methods for both Li^+-solvent and FSI^--solvent complexes is almost 1.0 with a very low mean absolute deviation (MAE) of 0.85 kcal mol^{-1} and 0.43 kcal mol^{-1}, respectively.

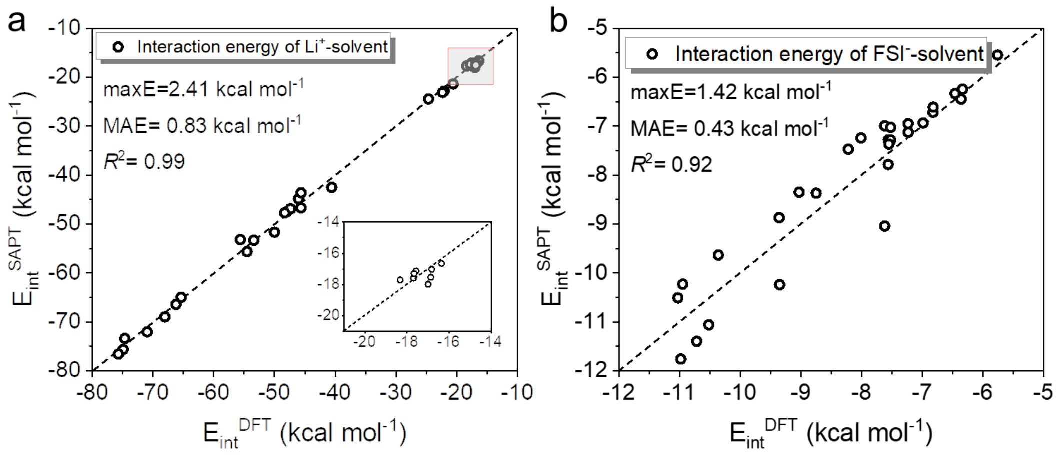

Supplementary Fig. 3 Comparison between the interaction energy of studied dimer complexes computed at the DFT and SAPT method. a Li^+-slvent interactions, **b**, FSI^--solvent interactions. The linear correlation coefficient R^2 between the interaction energies evaluated at the two different methods for both Li^+-solvent and FSI^--solvent complexes is almost 1.0 with a very low mean absolute deviation (MAE) of 0.85 kcal mol^{-1} and 0.43 kcal mol^{-1}, respectively.

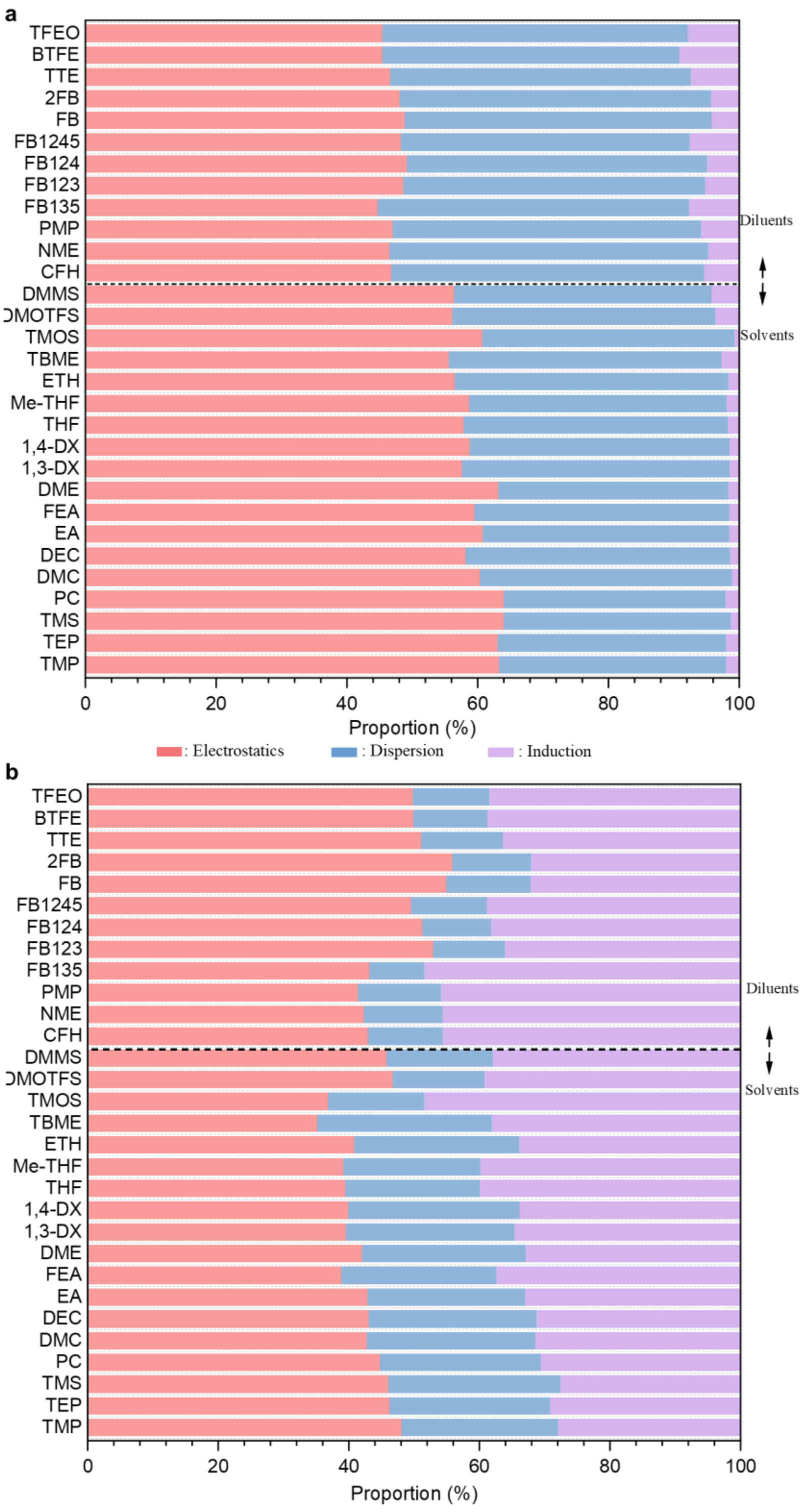

Supplementary Fig. 4 Contribution percentage plot of the SAPT-derived constructive electrostatic (E_{ele}), induction (E_{ind}), and dispersion (E_{dis}) interactions. a, between FSI^- and molecules. **b**, between Li^+ and molecules. The van der Waals force ratio is obtained by SAPT analysis.

Supplementary Note 1

In the framework of the SAPT approach, the interaction energy (E_{int}) can be decomposed into various physically meaningful components and can be expressed as $E_{int} = E_{ele} + E_{exc} + E_{ind} + E_{dis}$. The E_{ele} term mainly reflects the classical electrostatic interaction between the monomers. The E_{exc} term denotes the exchange-repulsion contribution caused by the overlap of the monomer wave function as well as the anti-symmetric requirements due to the fermionic behavior of the electrons in the dimer. The E_{ind} term portrays the induction contribution, which comprises polarization as the response of each monomer to the electric-field of the other one as well as charge transfer between two monomers. The E_{dis} term is the dispersion contribution due to the Coulomb correlation between electrons in one monomer with those in another one[1, 2]. The values given in Supplementary Tables 3, and 4 are converted into the contribution percentage for each attractive component, a more intuitive and deeper understanding could be obtained about the nature of the studied ion-solvent interactions. In this sense, the contribution percentage of each SAPT-derived attractive component was calculated for all of the studied complexes as $E_x/E_{ele} + E_{ind} + E_{dis} \times 100\%$, where E_x signifies E_{ele}, E_{ind}, or E_{dis}.

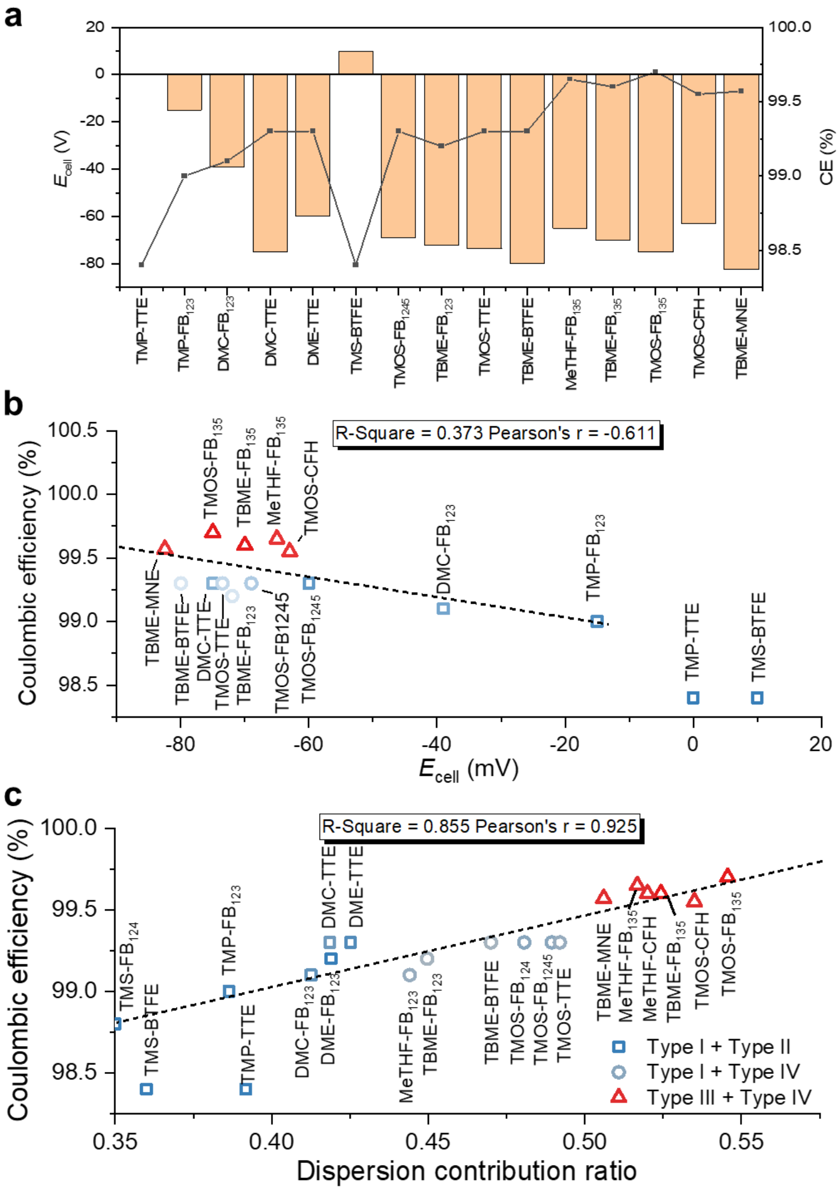

Supplementary Fig. 5 Solvation energy and dispersion contribution ratios of different high-performance electrolytes. a, Solvation energies of various electrolytes, **b**, Relationship of E_{cell} and CE, **c**, Relationship of dispersion contribution ratio and CE. The more negative the battery potential, the weaker the Li^+ solvation effect. The molecular structures with different symbols are shown in Supplementary Figure 1.

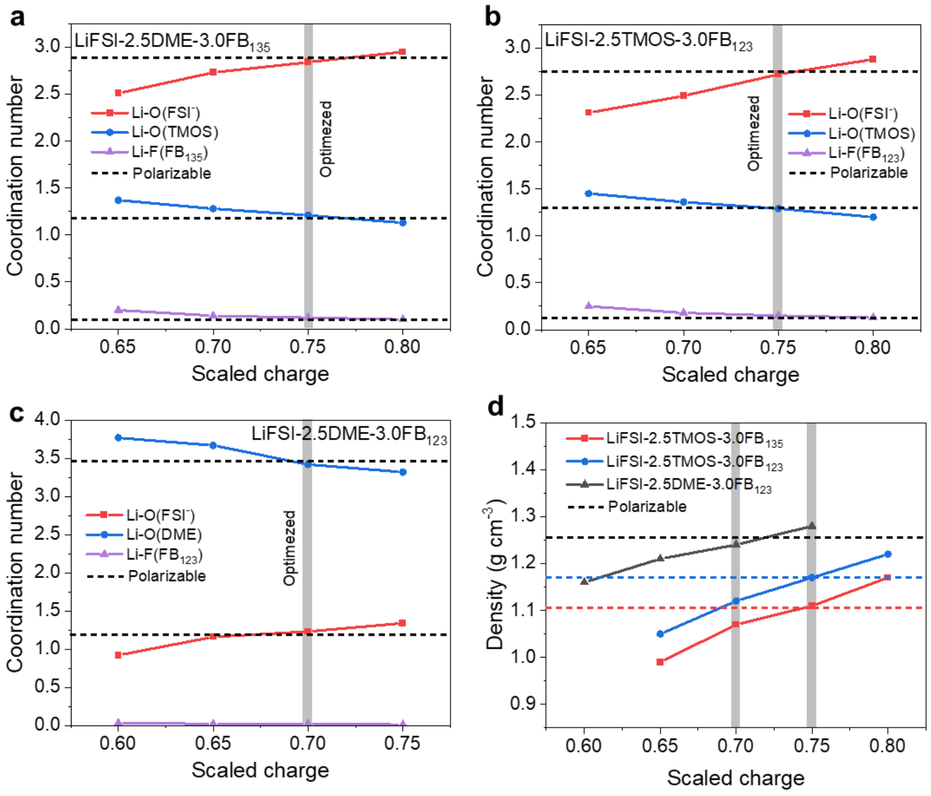

Supplementary Fig. 6 Electrolyte structure and physical parameters benchmark for various molecular dynamics protocols. **a-c**, Li coordination number, **d**, electrolyte density. The cutoff for the Li^+ solvation shell is set to 2.8 Å.

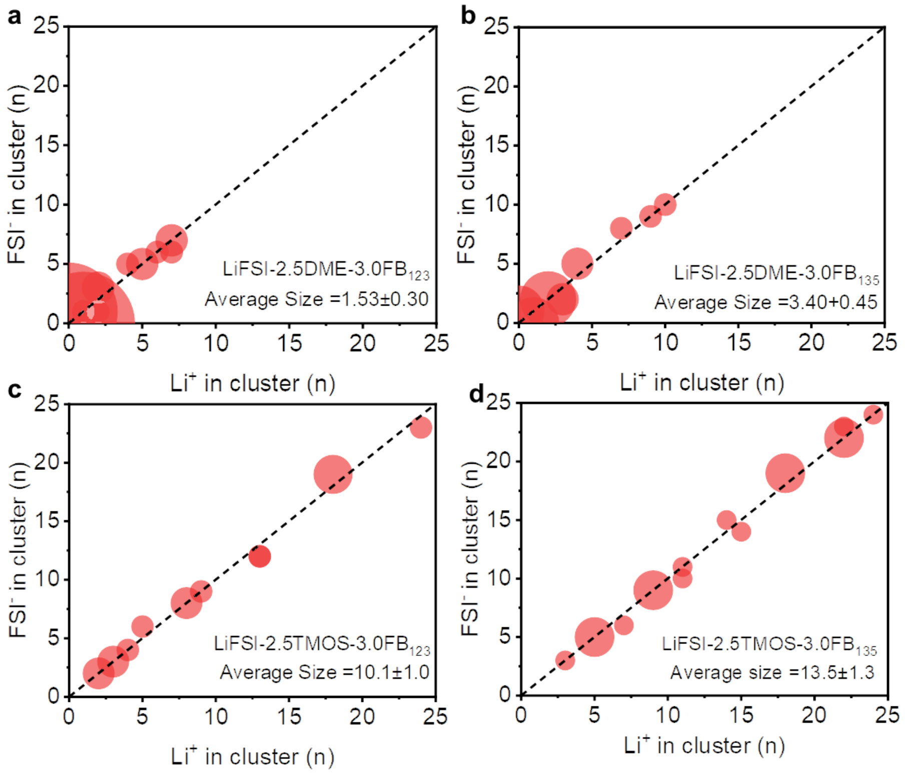

Supplementary Fig. 7 Size distribution of solvation clusters in different electrolytes. a, LiFSI-2.5DME-3.0FB$_{123}$. **b**, LiFSI-2.5DME-3.0FB$_{135}$. **c**, LiFSI-2.5TMOS-3.0FB$_{123}$. **d**, LiFSI-2.5TMOS-3.0FB$_{135}$. Larger bubbles mean higher probability of the corresponding cluster configuration. The parameter for average size is defined as the average number of Li^+ cations in the solvation cluster. The error is defined as the difference between the maximum and minimum in the time-averaged values in MD simulation.

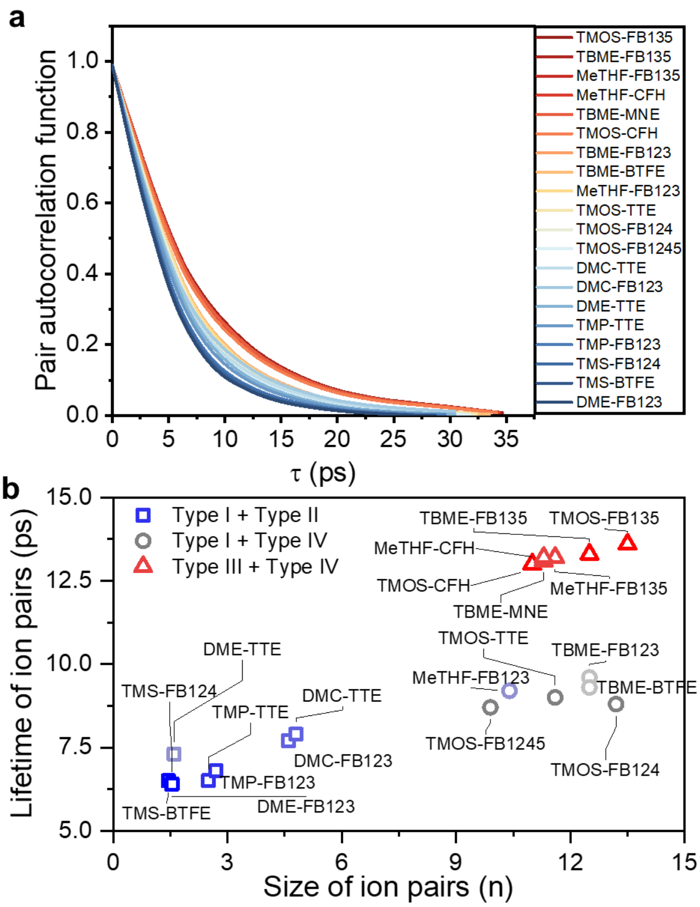

Supplementary Fig. 8. Statistical Li-FSI$^-$ ion pair lifetimes. **a** Li-FSI$^-$ pair existence autocorrelation function of different electrolytes with a cut-off distance of 2.5 Å. **b** Solvation size and corresponding Li-FSI$^-$ ion pair lifetime in various electrolytes. For judging the lifetime of Li-FSI$^-$ ion pair, the existence autocorrelation function was calculated with a cut-off distance of 2.5 Å between Li^+ and FSI^-. The total existence autocorrelation function is an average of all the different Li-FSI$^-$ ion pairs, which describes the time of the given criteria being fulfilled. The size of ion pairs is defined as the average number of Li^+ cations in the solvation cluster.

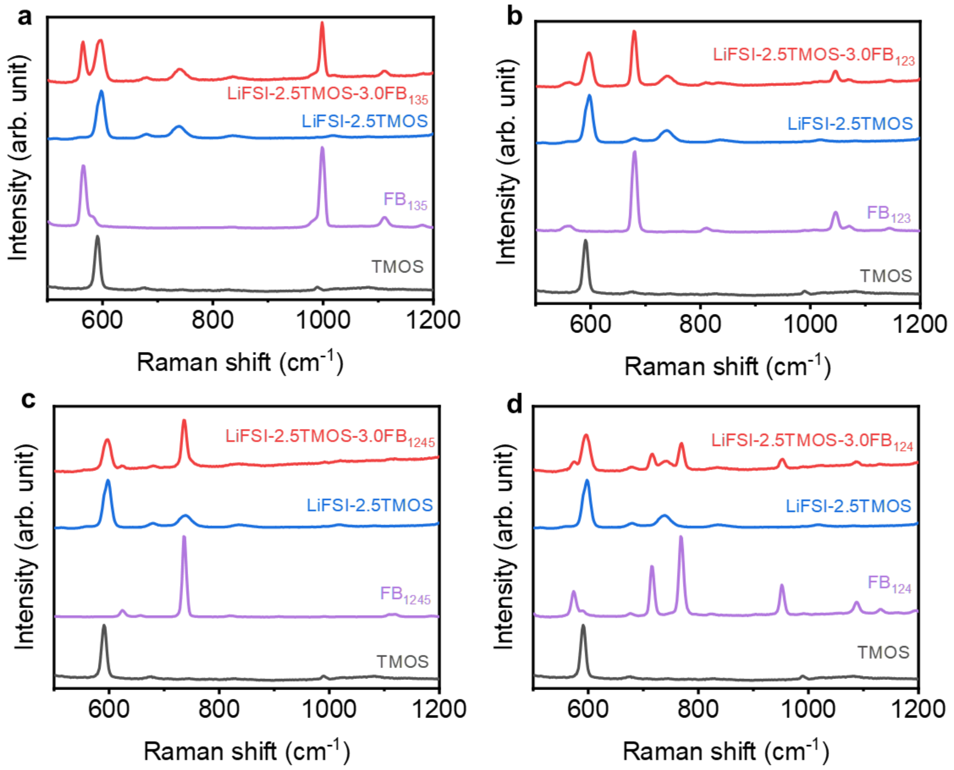

Supplementary Fig. 9 Raman spectra of different electrolytes. a, LiFSI-2.5TMOS-3.0FB$_{135}$. **b**, LiFSI-2.5TMOS-3.0FB$_{123}$. **c**, LiFSI-2.5TMOS-3.0FB$_{1245}$. **d**, LiFSI-2.5TMOS-3.0FB$_{124}$.

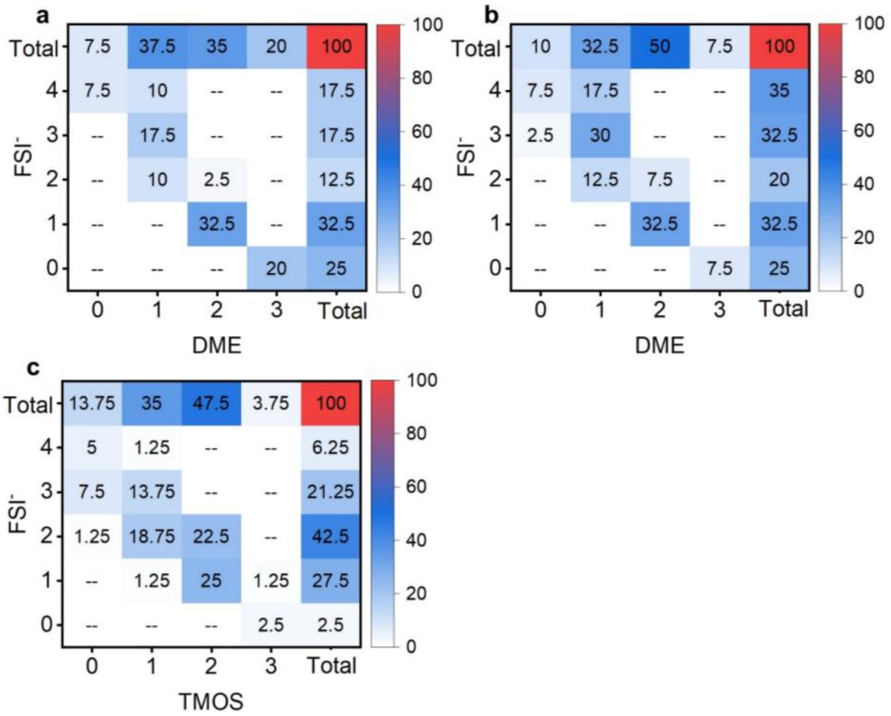

Supplementary Fig. 10 Heatmap showing the proportion of corresponding solvation structure for different electrolytes. a, LiFSI-2.5DME-3.0FB$_{123}$. **b**, LiFSI-2.5DME-3.0FB$_{135}$. **c**, LiFSI-2.5TMOS-3.0FB$_{123}$.

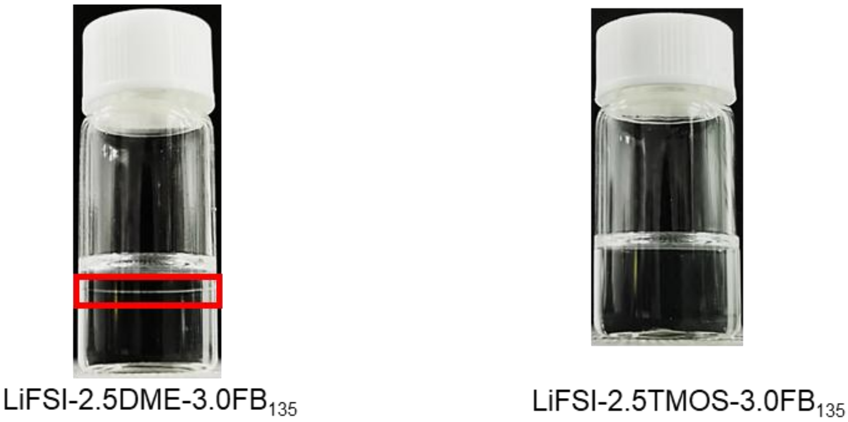

Supplementary Fig. 11 Digital photographs of two electrolytes. The phase boundary is highlighted with a red border.

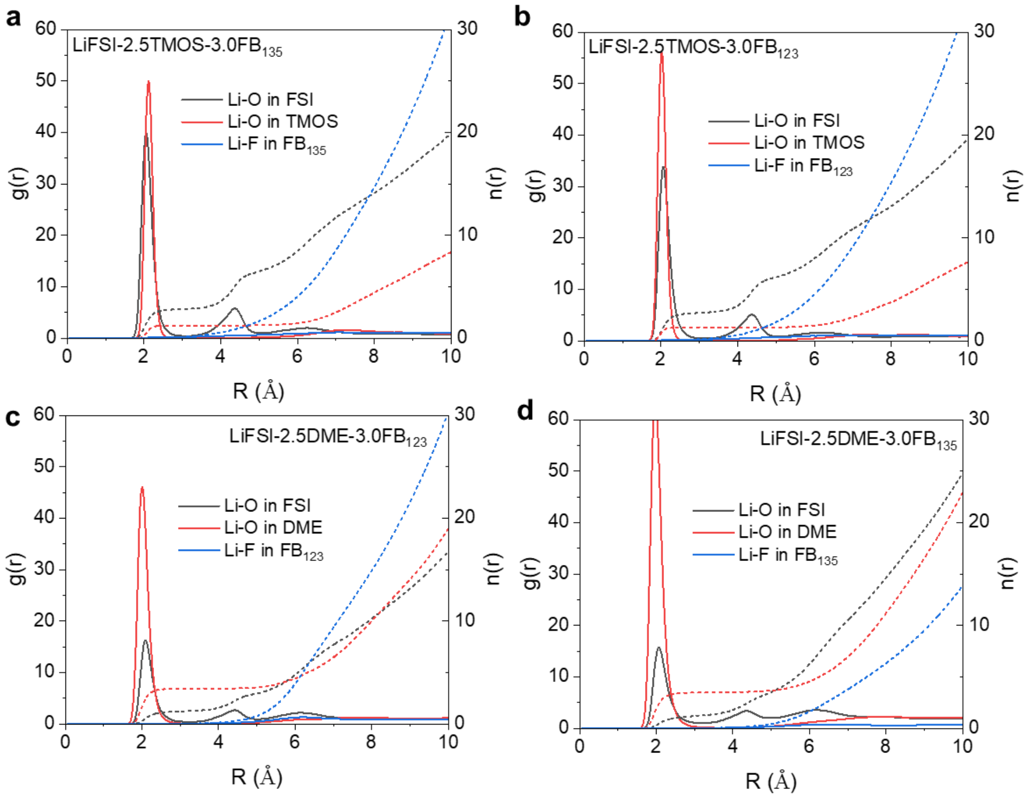

Supplementary Fig. 12 Radial distribution function (RDF) and coordination number (CN) plots for different electrolytes obtained by optimized parameters for classical force fields. a LiFSI-2.5TMOS-3.0FB$_{135}$, **b** LiFSI-2.5TMOS-3.0FB$_{123}$, **c** LiFSI-2.5DME-3.0FB$_{123}$, **d** LiFSI-2.5DME-3.0FB$_{135}$. RDF and CN as functions of distances between Li^+ and O, O and F atoms in FSI^-, DME, and FB molecules.

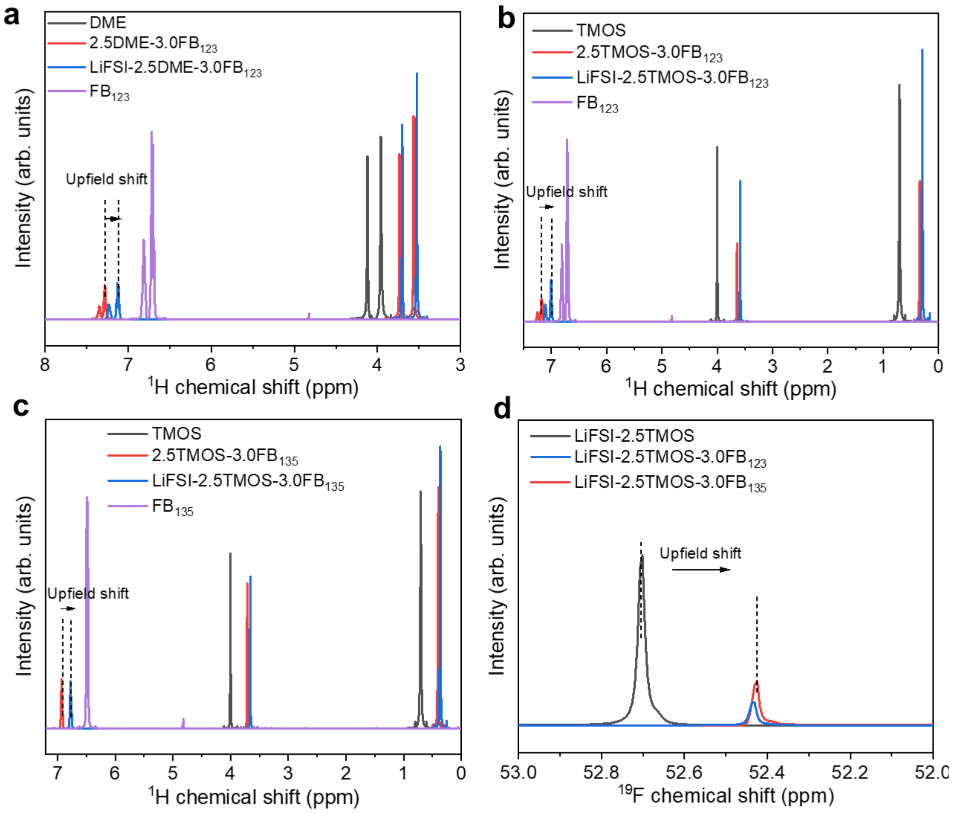

Supplementary Fig. 13 Intermolecular interaction analysis in different electrolytes. ^{1}H NMR spectra of various electrolytes: **a** LiFSI-2.5DME-3.0FB_{123}, **b** LiFSI-2.5TMOS-3.0FB_{123}, **c** LiFSI-2.5TMOS-3.0FB_{135}. **d**, ^{19}F NMR spectra of TMOS-based electrolyte.

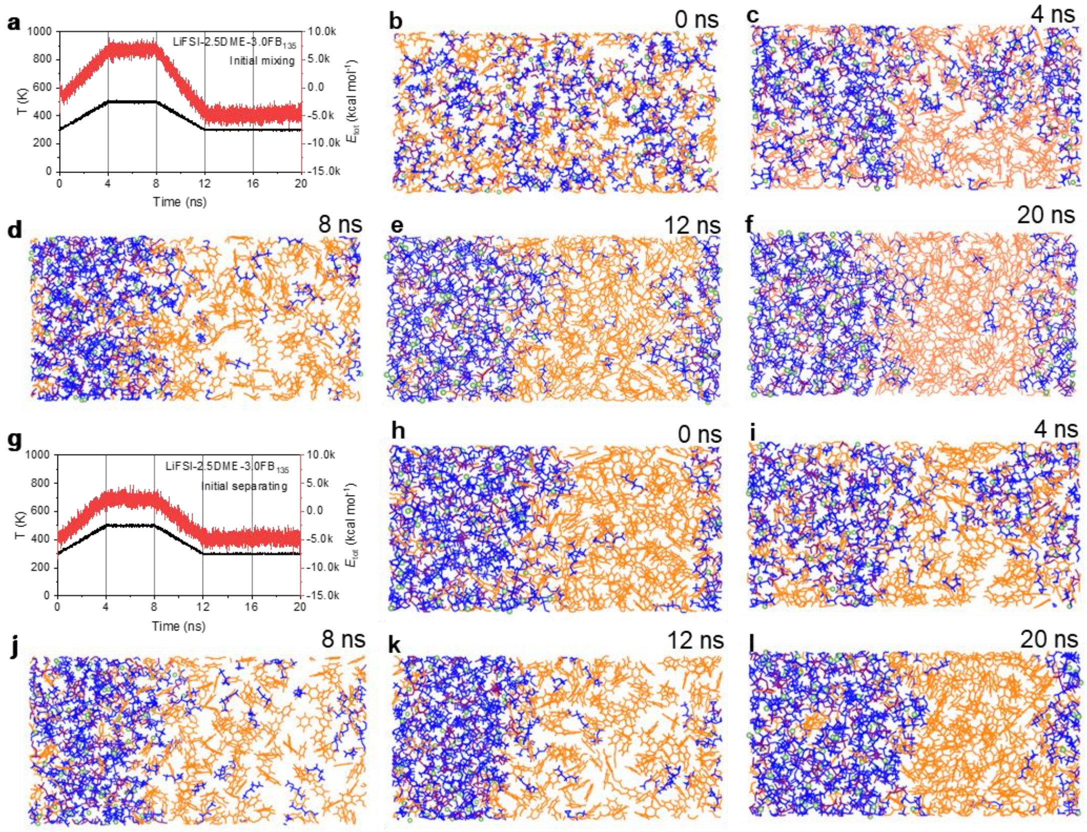

Supplementary Fig. 14 MD simulations of LiFSI-2.5DME-3.0FB$_{135}$ starting from different initial configurations. a-f, Evolutions of total energies of MD simulations and snapshots at 0, 4, 8, 12, and 20 ns, starting with all LiFSI, DME, and FB$_{135}$ being randomly packed into the simulation box. **g-l**, Evolutions of total energies of MD simulations and snapshots at 0, 4, 8, 12, and 20 ns, starting with separating the LiFSI/DME phase from the FB$_{135}$ phase in the simulation box. In order to facilitate adequate mixing and reduce the computational cost of some steps, the temperature was set to increase from 300 K to 500 K within 4 ns, hold for 4 ns, then cool to 300 K within 4 ns, and then relax at 300 K for 8 ns. Colour code: green: Li cation, purple: FSI anion, FB$_{135}$: orange, blue: DME. The same algorithm was also adopted in **Supplementary Fig. 15**.

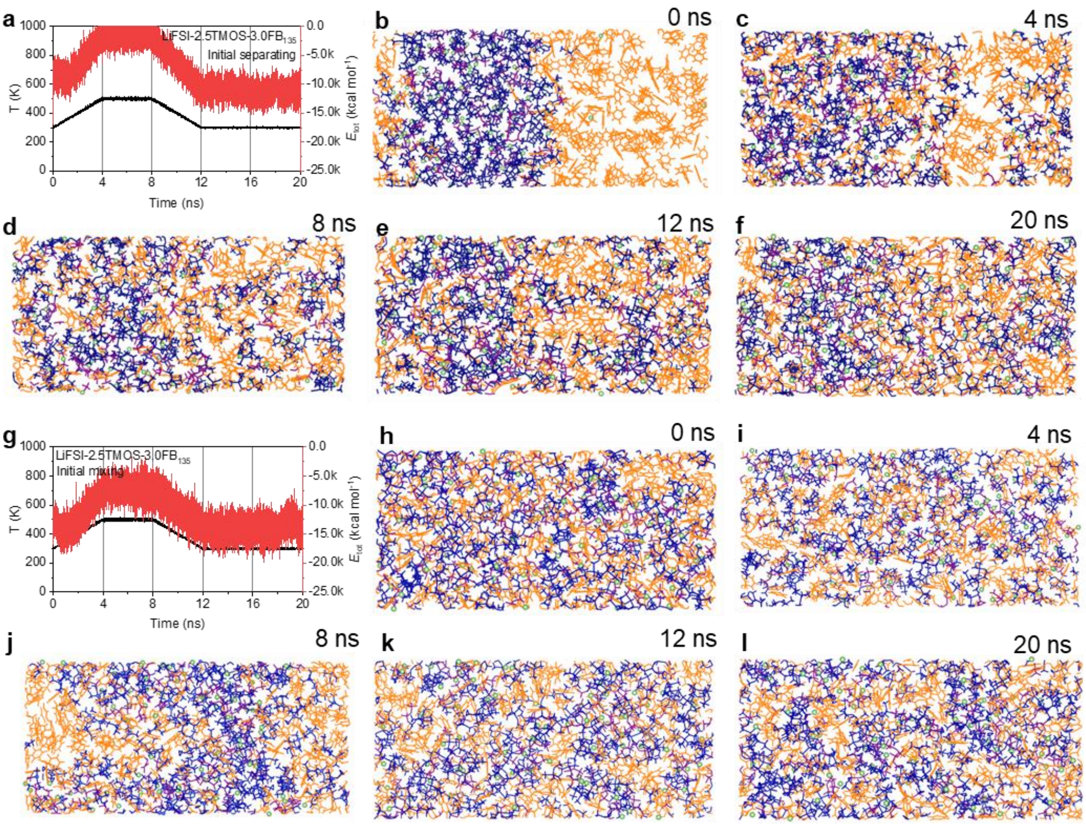

Supplementary Fig. 15 MD simulations of LiFSI-2.5TMOS-3.0FB$_{135}$ starting from different initial configurations. a-f, Evolutions of total energies of MD simulations and snapshots at 0, 4, 8, 12, and 20 ns, starting with separating the LiFSI/TMOS phase from the FB$_{135}$ phase in the simulation box. **g-l**, Evolutions of total energies of MD simulations and snapshots at 0, 4, 8, 12, and 20 ns, starting with all LiFSI, TMOS, and FB$_{135}$ being randomly packed into the simulation box. Colour code: green: Li cation, purple: FSI anion, FB$_{135}$: orange, blue: TMOS.

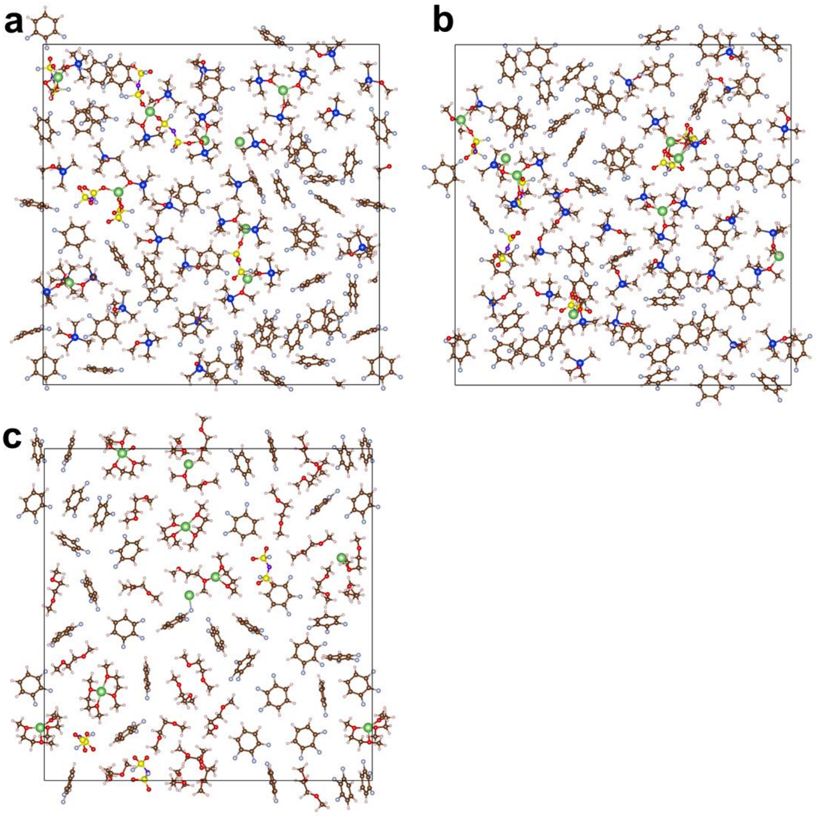

Supplementary Fig. 16. Snapshots of inner-Helmholtz interfacial regions of the negative elctrode surface. a, LiFSI-2.5TMOS-3.0FB$_{123}$. **b,** LiFSI-2.5TMOS-3.0FB$_{135}$. **c,** LiFSI-2.5DME-3.0FB$_{123}$. Colour code: green: Li atom, red: O atom, yellow: S atom, purple: N atom, cyan: F atom, Si: blue, tan: C atom, white: H atom.

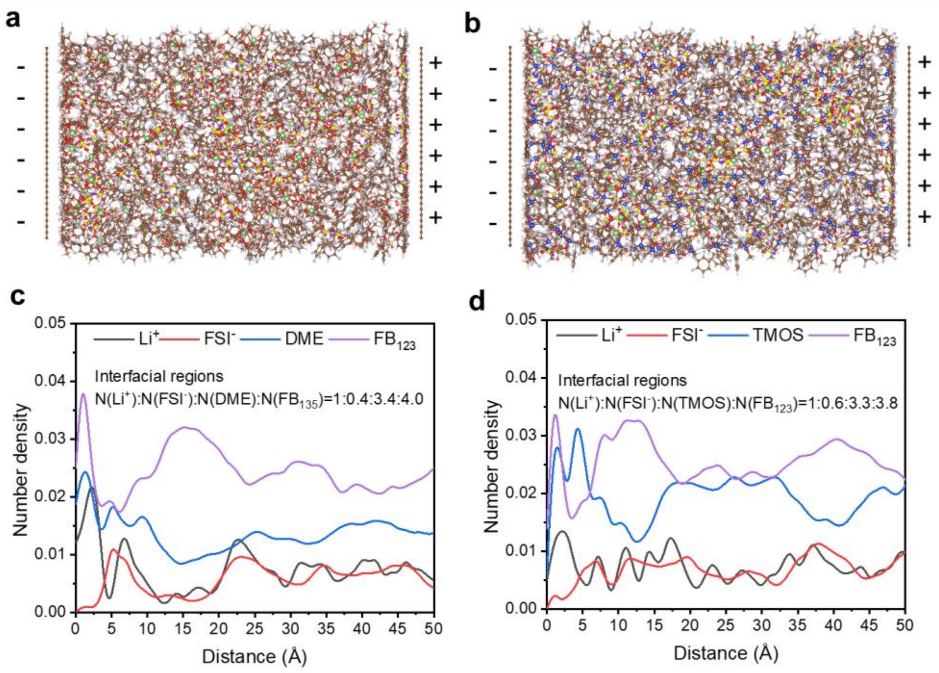

Supplementary Fig. 17. The snapshot and number density (ρ) profiles of ions and solvent molecules in different electrolytes. a, c LiFSI-2.5DME-3.0FB_{123}. **b, d** LiFSI-2.5TMOS-3.0FB_{123}. Colour code: green: Li atom, red: O atom, yellow: S atom, purple: N atom, cyan: F atom, Si: blue, tan: C atom, white: H atom.

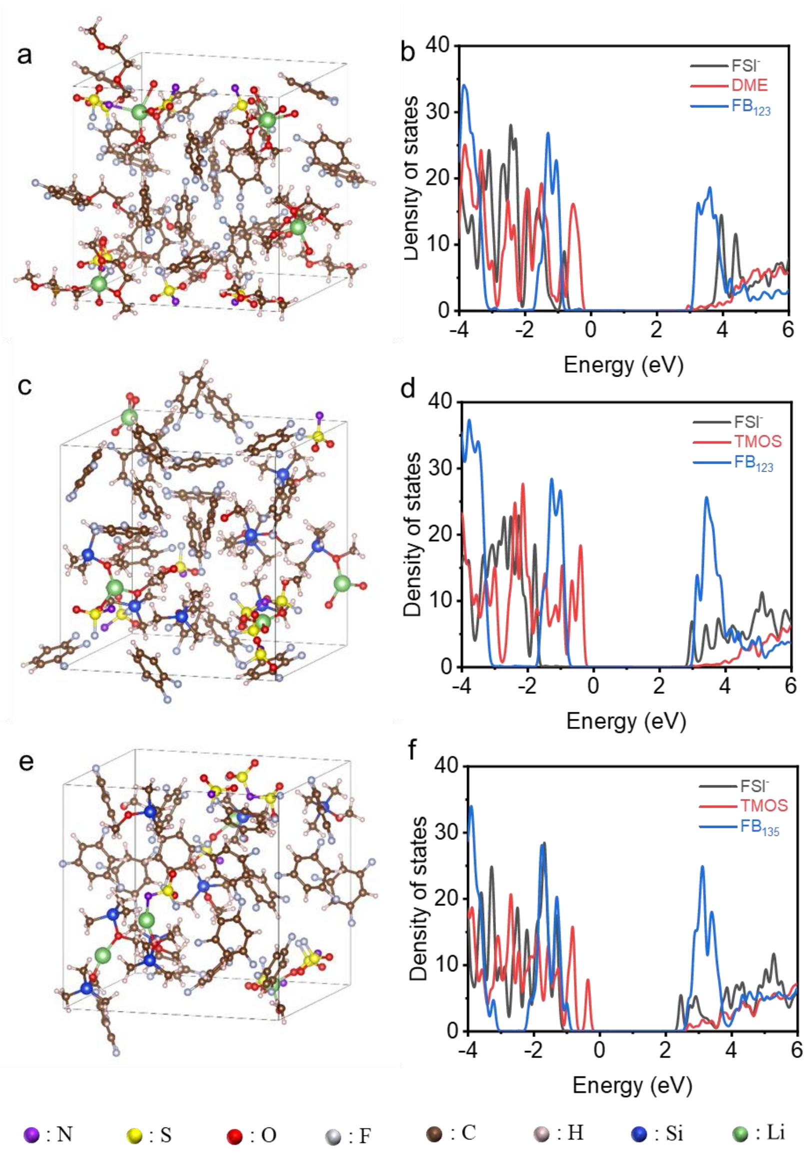

Supplementary Fig. 18 The snapshots and projected density of states (PDOS) plots of different electrolytes. a, b LiFSI-2.5DME-3.0FB_{123}. **c, d** LiFSI-2.5TMOS-3.0FB_{123}. **e, f** LiFSI-2.5TMOS-3.0FB_{135}. Colour code: green: Li atom, red: O atom, yellow: S atom, purple: N atom, cyan: F atom, Si: blue, tan: C atom, white: H atom.

.

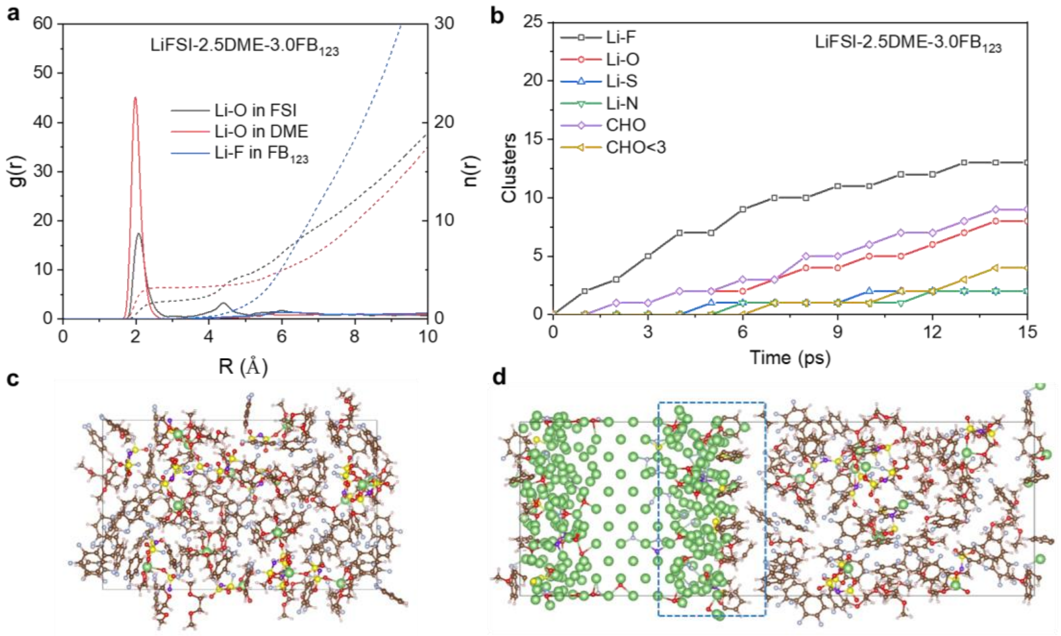

Supplementary Fig. 19 AIMD simulations for LiFSI-2.5DME-3.0FB$_{123}$ electrolyte on Li metal. a, Radical distribution functions calculated from initial structure before reaction with Li metal, **b,** Evolution of reaction products during AIMD simulation, **c**, Snapshot revealing the initial structure before reaction with Li metal, **d**, Snapshot revealing the chemical reactions at the Li metal-electrolyte interface. Colour code: green: Li atom, red: O atom, yellow: S atom, purple: N atom, cyan: F atom, Si: blue, tan: C atom, white: H atom.

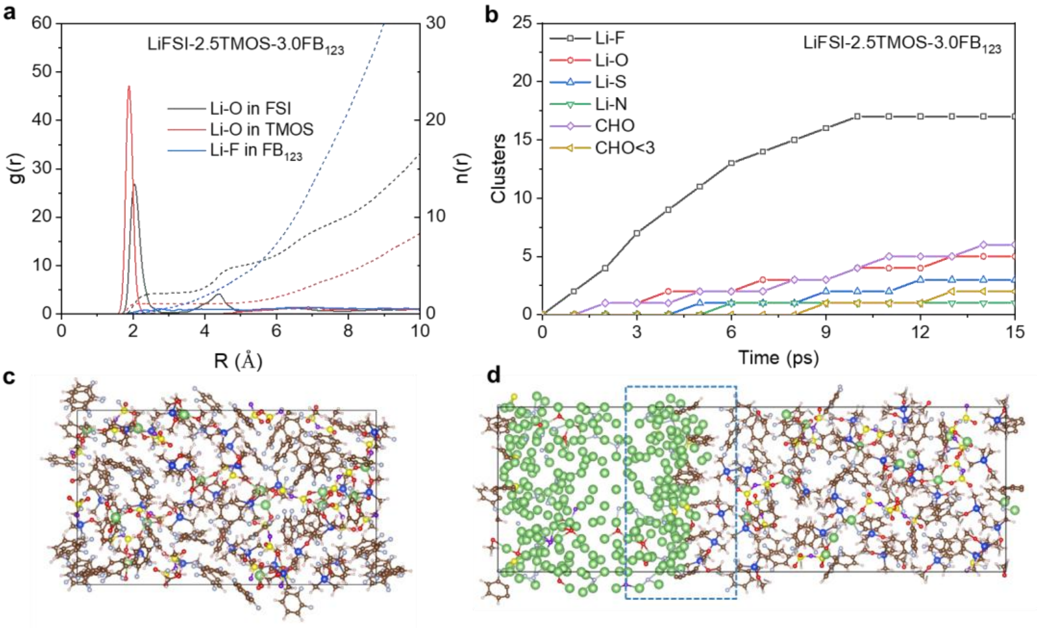

Supplementary Fig. 20 AIMD simulations for LiFSI-2.5TMOS-3.0FB$_{123}$ electrolyte on Li metal. a, Radical distribution functions calculated from initial structure before reaction with Li metal, **b,** Evolution of reaction products during AIMD simulation, **c**, Snapshot revealing the initial structure before reaction with Li metal, **d**, Snapshot revealing the chemical reactions at the Li metal-electrolyte interface. Colour code: green: Li atom, red: O atom, yellow: S atom, purple: N atom, cyan: F atom, Si: blue, tan: C atom, white: H atom.

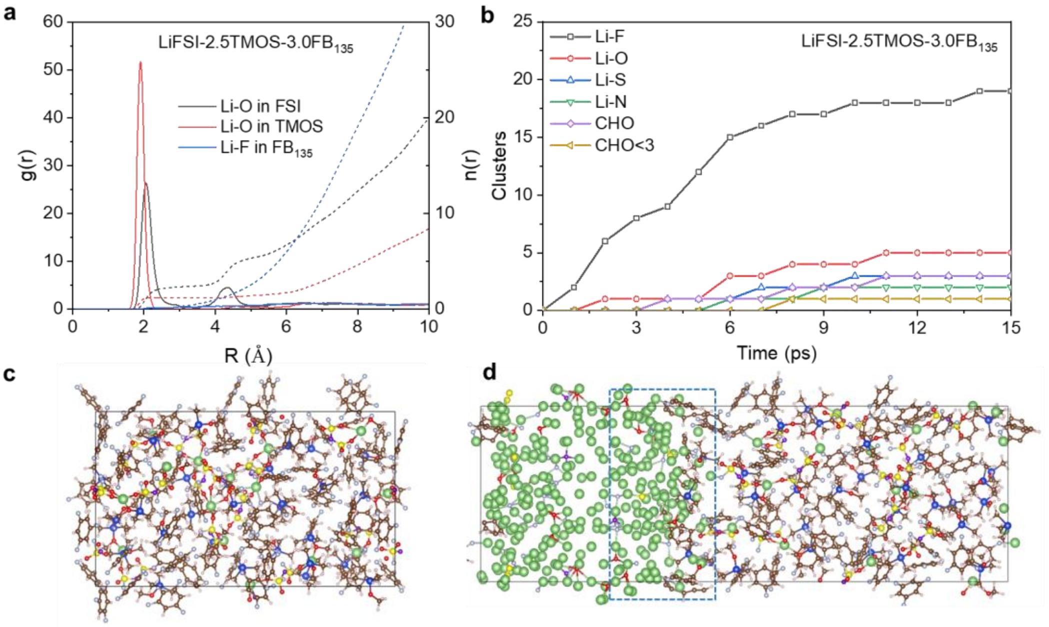

Supplementary Fig. 21 AIMD simulations for LiFSI-2.5TMOS-3.0FB$_{135}$ electrolyte on Li metal. a, Radical distribution functions calculated from initial structure before reaction with Li metal, **b,** Evolution of reaction products during AIMD simulation, **c**, Snapshot revealing the initial structure before reaction with Li metal, **d**, Snapshot revealing the chemical reactions at the Li metal-electrolyte interface. Colour code: green: Li atom, red: O atom, yellow: S atom, purple: N atom, cyan: F atom, Si: blue, tan: C atom, white: H atom.

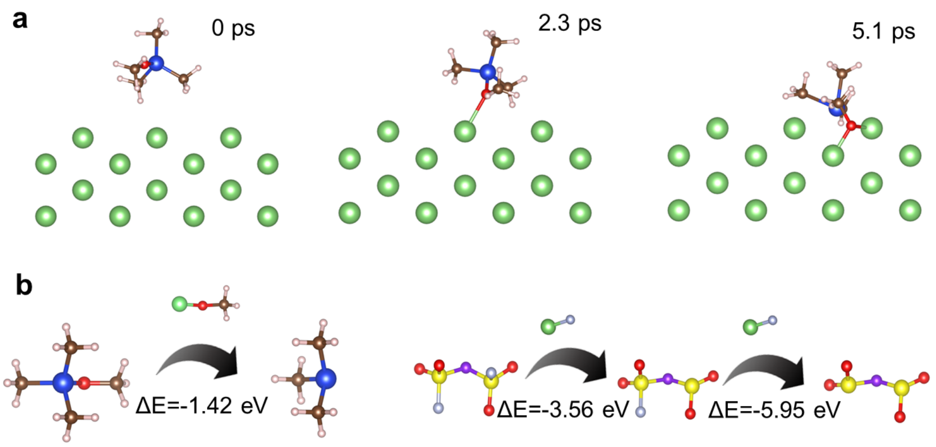

Supplementary Fig. 22 Decomposition simulation and energetics for TMOS and FSI$^-$ anion. **a**, TMOS decomposition simulation on Li surface. **b**, energetics for multistep decomposition pathways of TMOS and FSI$^-$ anion. Colour code: green: Li atom, red: O atom, yellow: S atom, purple: N atom, cyan: F atom, Si: blue, tan: C atom, white: H atom.

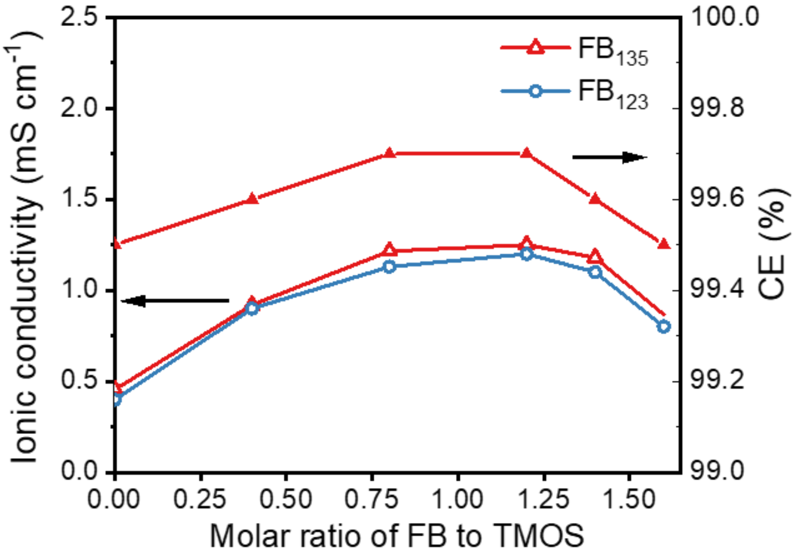

Supplementary Fig. 23 Ionic conductivity and CE of electrolytes with different FB:TMOS ratios.

Supplementary Note 2

CE uncertainty was calculated by finding the error in current measurement from the LAND using a current equivalent to that used in Li plating and stripping experiments (0.5 mA cm^{-2}). Current measurements were made through a calibrated ammeter and a shunt resistor in series with the LAND. Errors on several LAND channels were measured. These come out to be a 0.20% underestimation of the measured efficiency, which are rounded to measurement uncertainties of ±0.2%.

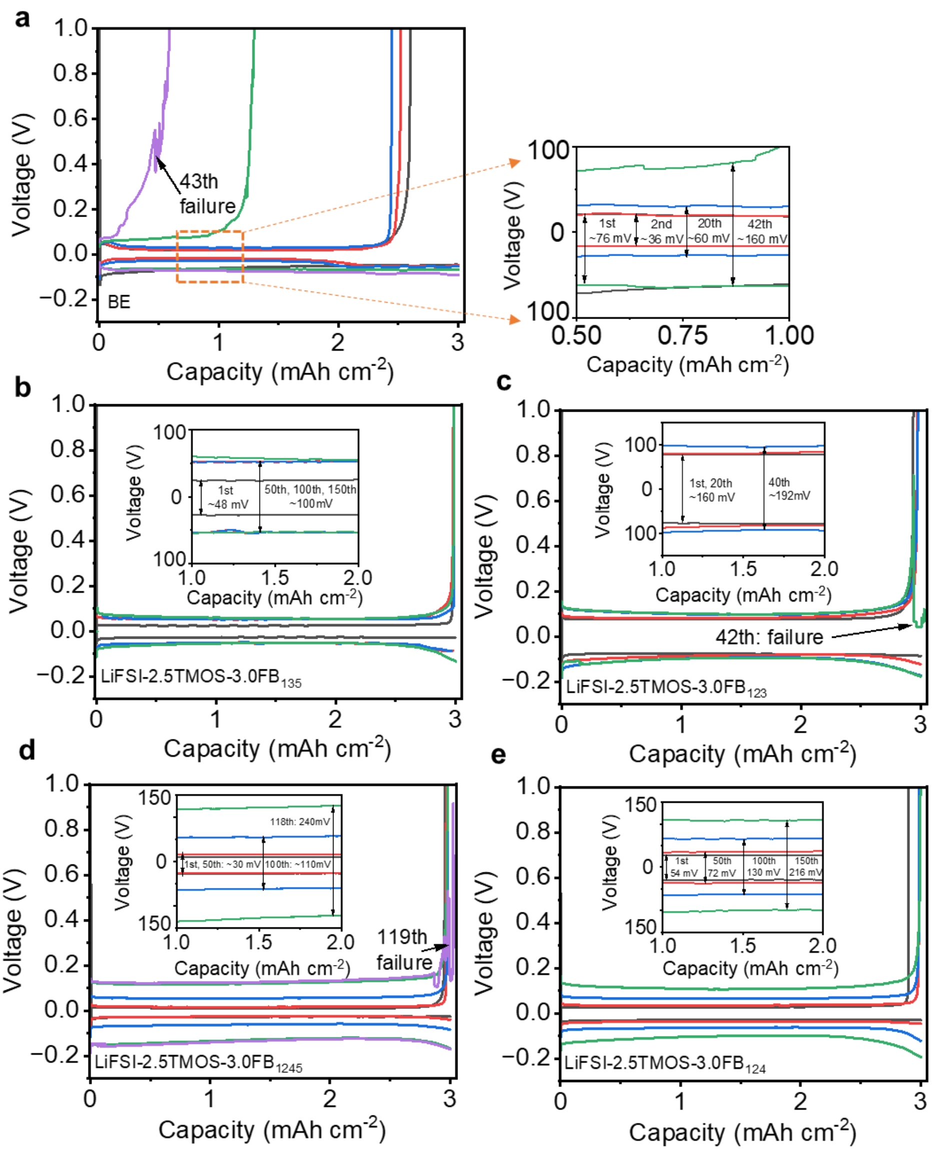

Supplementary Fig. 24 Polarization of Li metal plating/stripping in different electrolytes. a, BE. **b**, LiFSI-2.5TMOS-3.0FB$_{135}$, **c**, LiFSI-2.5TMOS-3.0FB$_{123}$. **d**, LiFSI-2.5TMOS-3.0FB$_{1245}$. **e**, LiFSI-2.5TMOS-3.0FB$_{124}$.

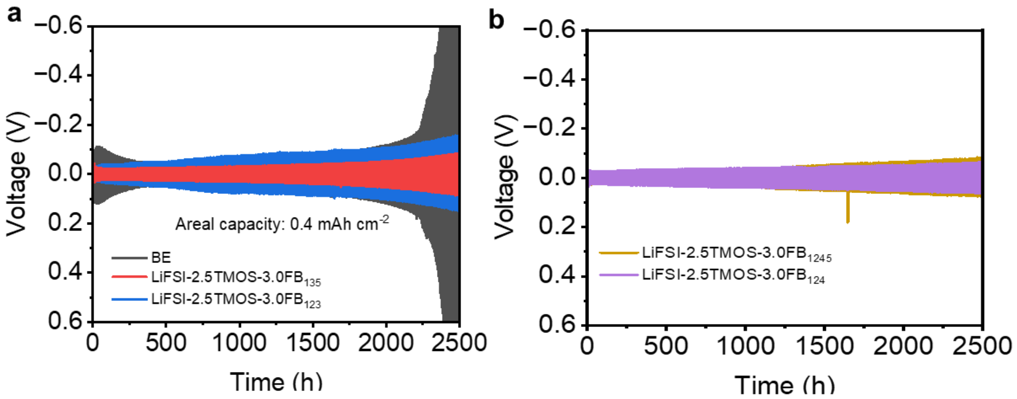

Supplementary Fig. 25 Long-term cycling stability of Li||Li cells for different electrolytes (0.2 mA cm^{-2}, 0.4 mAh cm^{-2}).

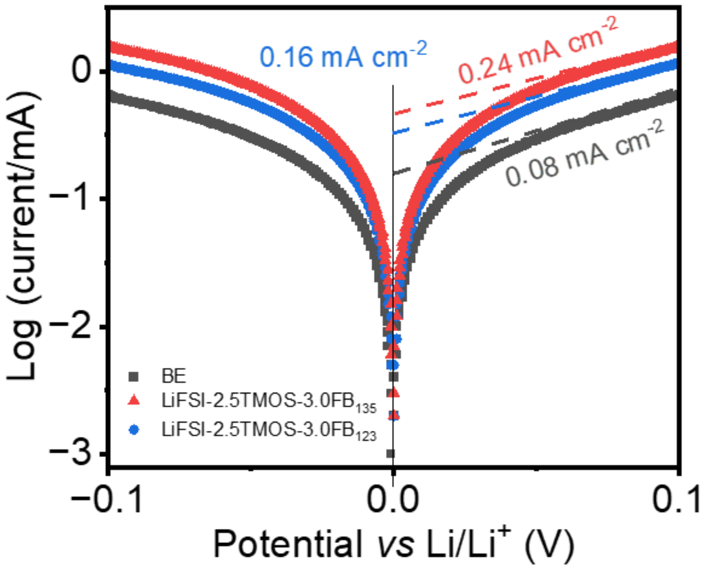

Supplementary Fig. 26 Tafel plots of Li plating/stripping in different electrolytes.

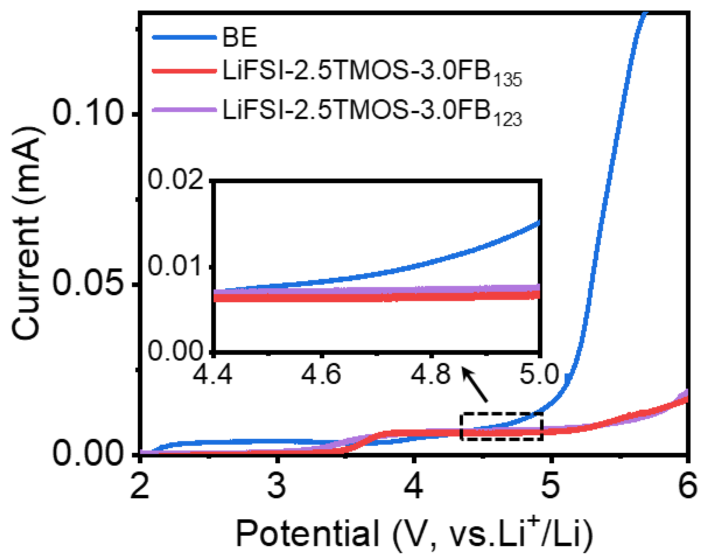

Supplementary Fig. 27 Linear sweep voltammetry curves of different electrolytes obtained from Li||Al half-cells at a scan rate of 1.0 mV s^{-1} from 2.0 to 6.0 V.

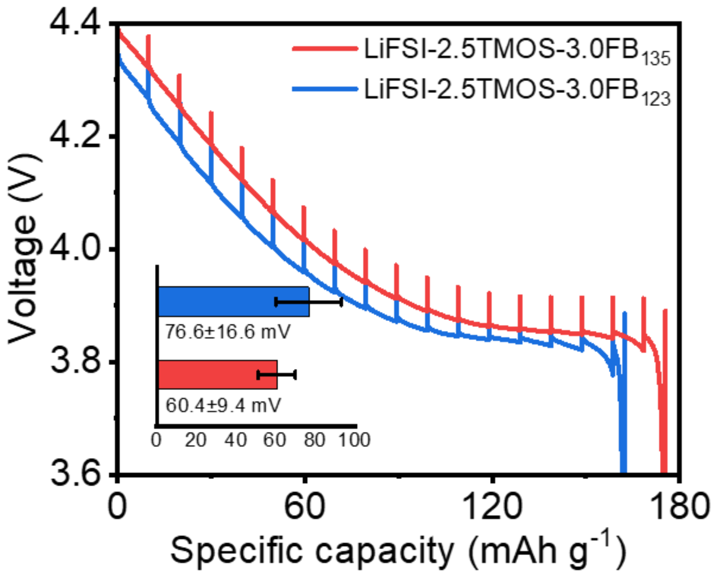

Supplementary Fig. 28 Discharge voltage curves obtained from GITT measurements at 0.33C.

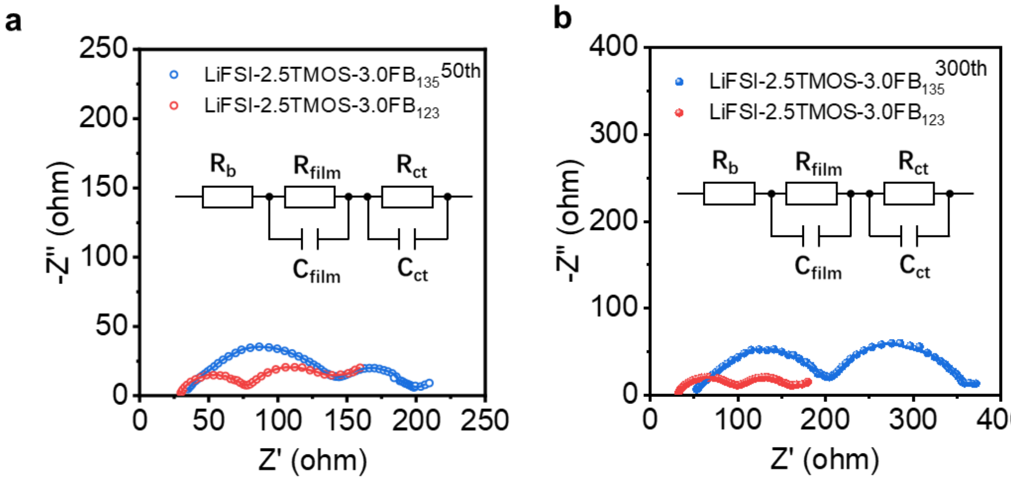

Supplementary Fig. 29 Nyquist plots of 20 µm-Li||LCO cells with different electrolytes. a, after 50 cycles. **b**, after 300 cycles. The raw impedance data are reported as symbols, the fitted data are reported in line form and the inset is the equivalent circuit.

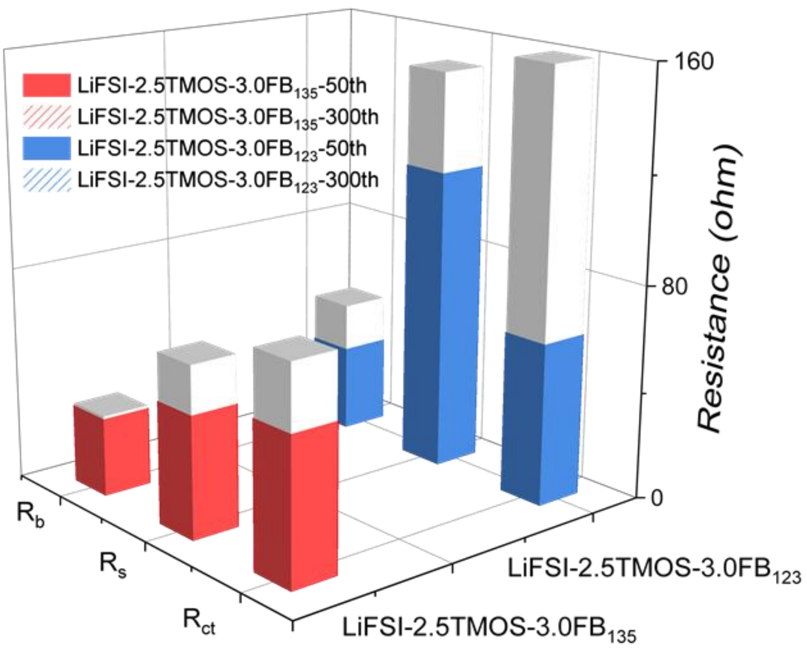

Supplementary Fig. 30 Evolution of resistance of 20 μm-Li||LCO cells with different electrolytes.

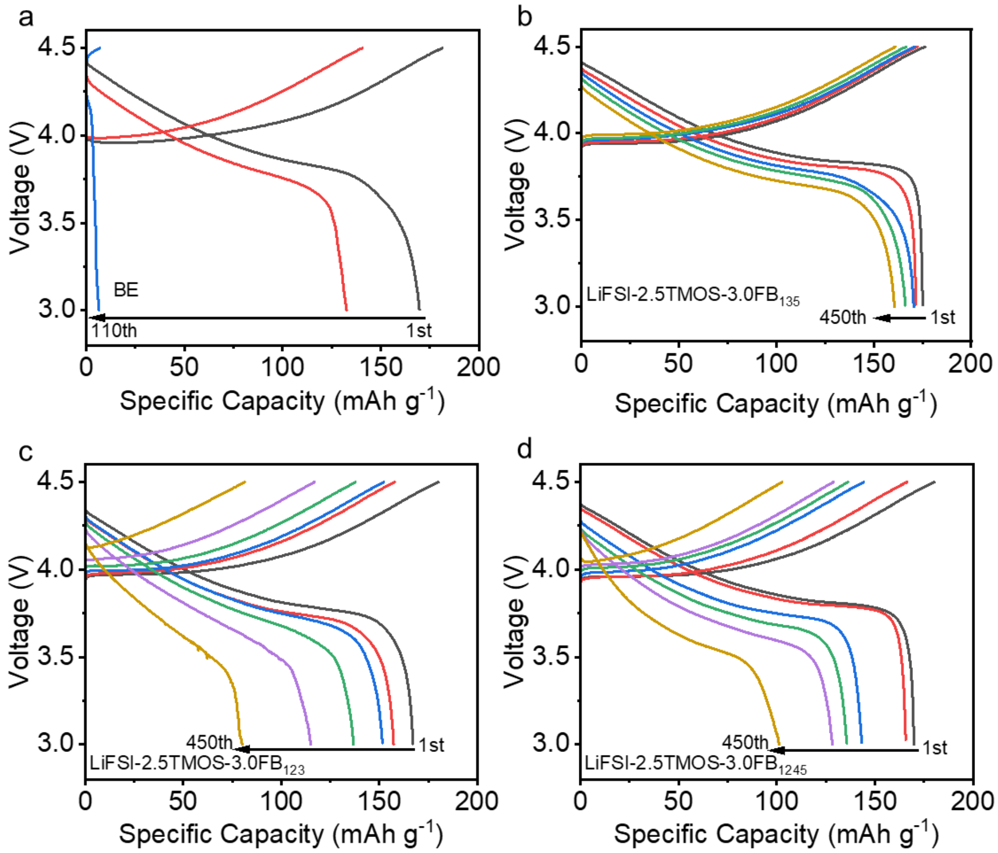

Supplementary Fig. 31 Voltage profiles of 20 μm-Li||4.5 V-LCO cells with different electrolytes.

a, BE. **b**, LiFSI-2.5TMOS-3.0FB$_{135}$. **c**, LiFSI-2.5TMOS-3.0FB$_{123}$. **d**, LiFSI-2.5TMOS-3.0FB$_{1245}$.

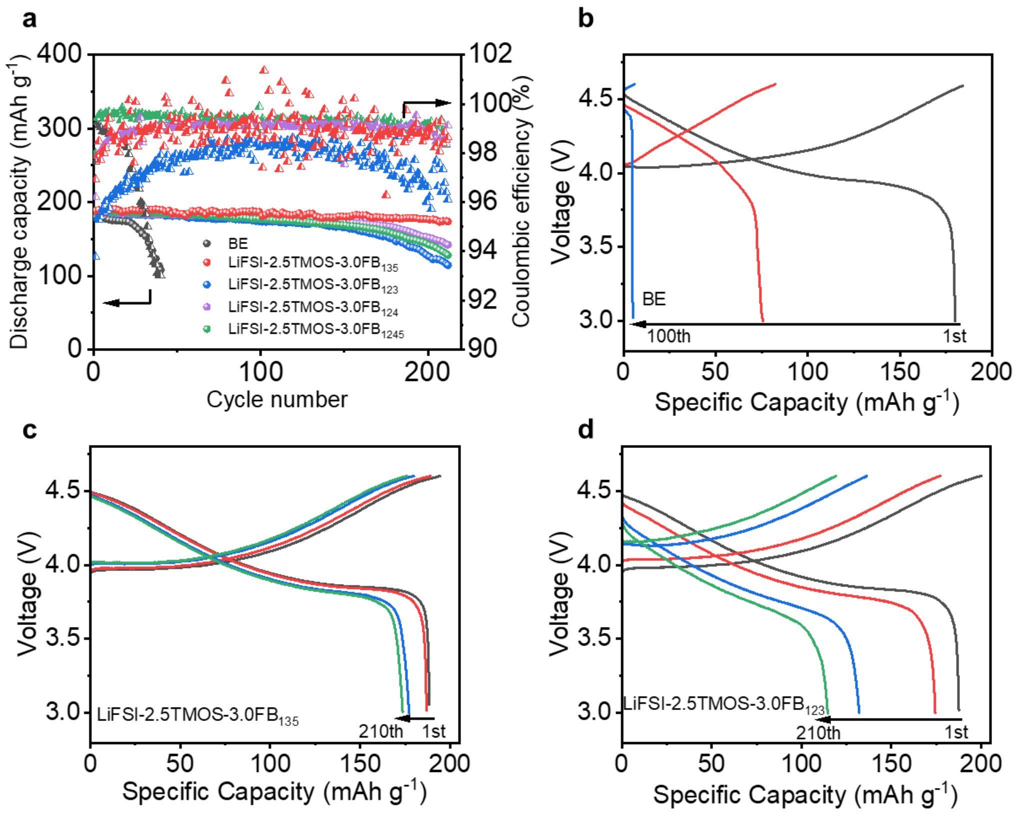

Supplementary Fig. 32 Electrochemical performances of 20 μm-Li||4.6 V-LCO cells in different electrolytes. a, Discharge specific capacity and Coulombic efficiency during long-term cycling at 0.2C/0.5C charge/discharge (LCO loading: 2.0 mAh cm^{-2}, 1C = 180 mA g^{-1}). **b-d**, Corresponding voltage profiles in different electrolytes: **b**, BE. **c**, LiFSI-2.5TMOS-3.0FB_{135}. **d**, LiFSI-2.5TMOS-3.0FB_{123}.

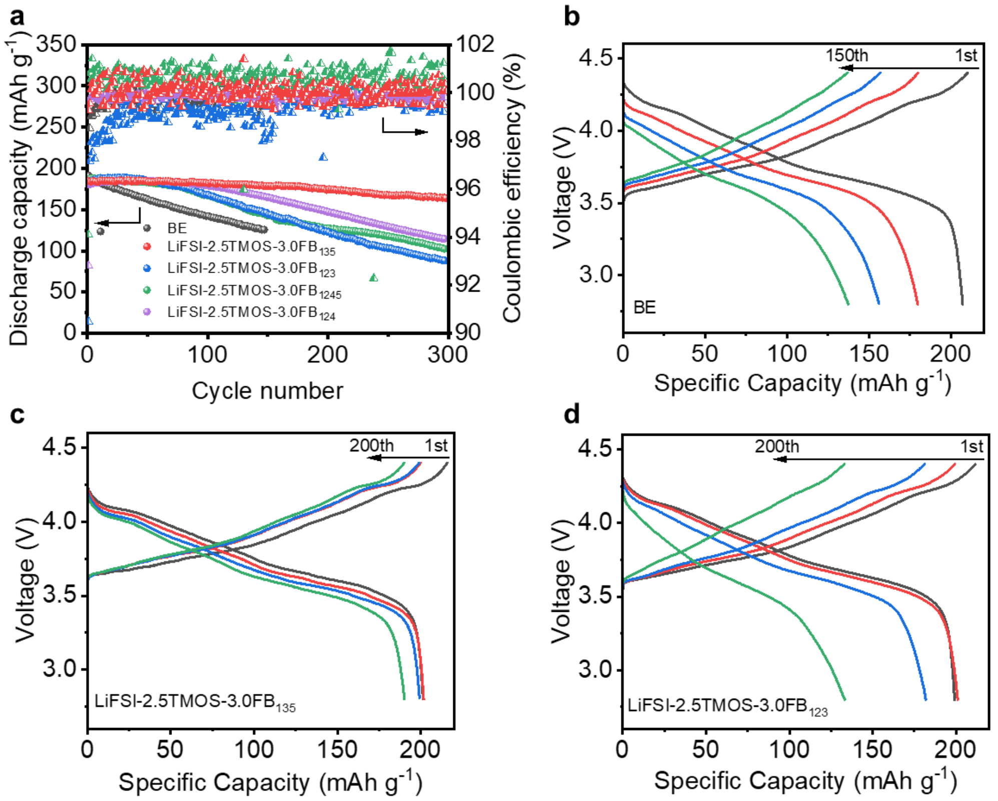

Supplementary Fig. 33 Electrochemical performances of 20 μm-Li||4.4 V-NCM811 cells with different electrolytes. a, Discharge specific capacity and Coulombic efficiency during long-term cycling at 0.2C/0.5C charge/discharge (NCM811 loading: 2.3 mAh cm^{-2}, 1C = 200 mA g^{-1}). **b-d**, Corresponding voltage profiles in different electrolytes: **b**, BE. **c**, LiFSI-2.5TMOS-3.0FB_{135}. **d**, LiFSI-2.5TMOS-3.0FB_{123}.

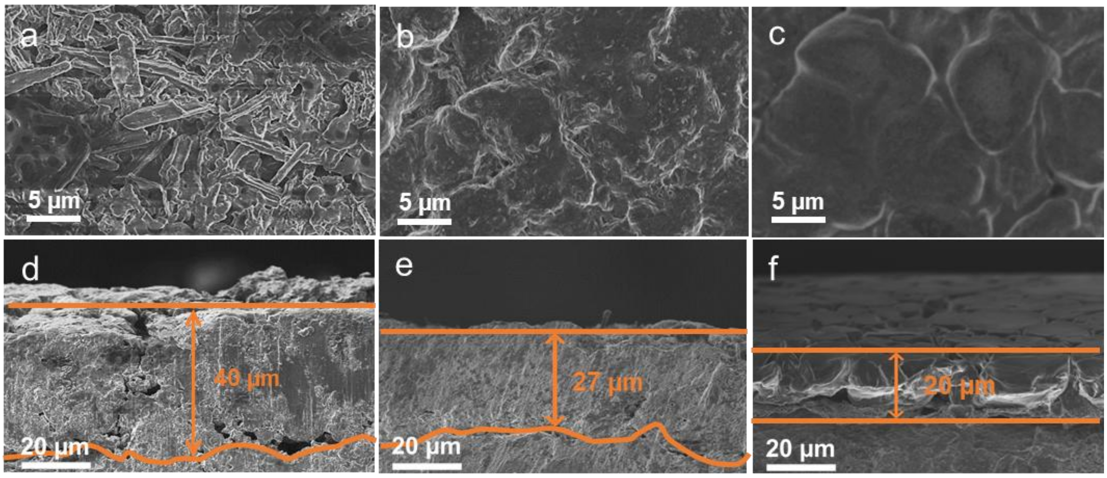

Supplementary Fig. 34 Morphology of cycled Li metal negative electrodes from Li||Li cells after 20 cycles at 25 °C (0.5 mA cm^2, 4 mAh cm^2) using different electrolytes. Top views (a-c) and cross-section view (d-f) BE (a and d), LiFSI-2.5TMOS-3.0FB$_{123}$ (b and e) and LiFSI-2.5TMOS-3.0FB$_{135}$ (c and f).

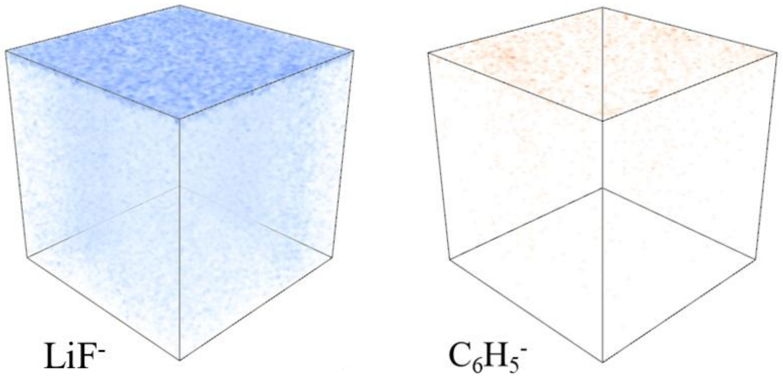

Supplementary Fig. 35 3D maps presenting the distribution of different fragments (LiF^-, $C_6H_5^-$) on the cycled Li metal negative electrodes from Li||Cu cells after 20 cycles (0.5 mA cm^{-2}, 4 mAh cm^{-2}) at 25 °C in LiFSI-2.5TMOS-3.0FB_{135}, sample area 50 × 50 µm with 600 s sputter time.

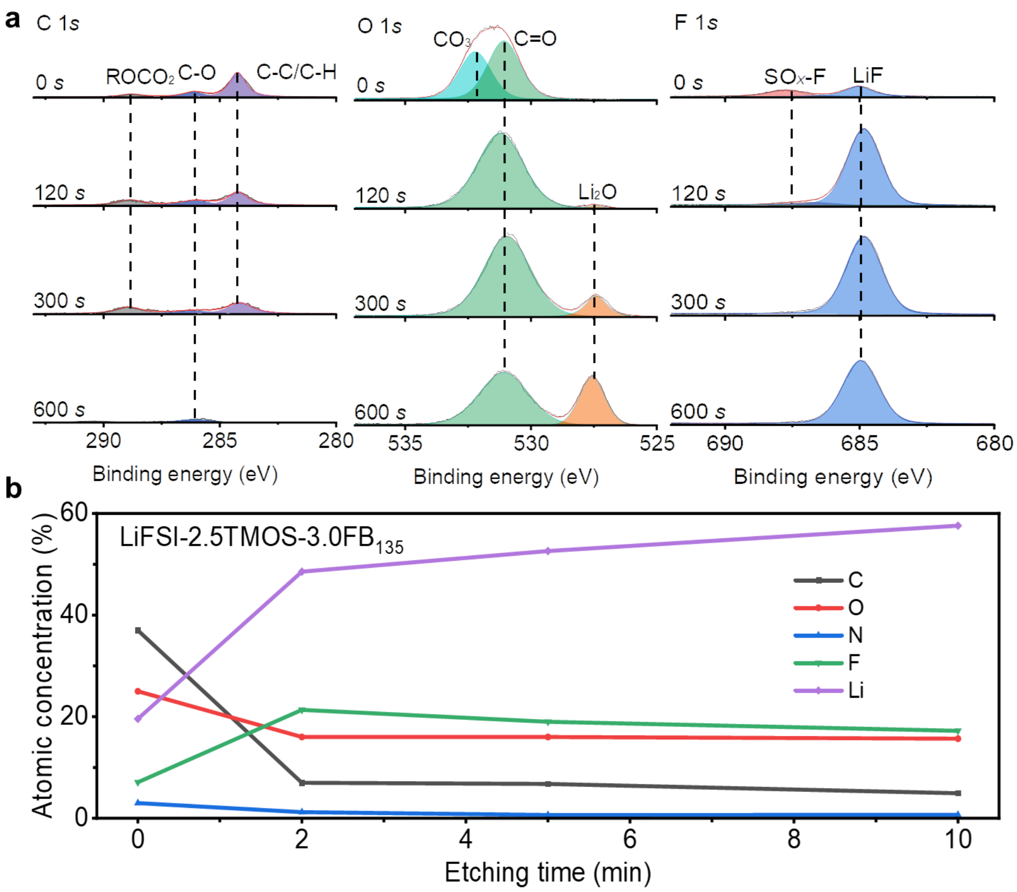

Supplementary Fig. 36 XPS characterization of SEIs formed on cycled Li metal negative electrodes after 10 cycles (0.5 mA cm^2, 4 mAh cm^2) at 25 °C in LiFSI-2.5TMOS-3.0FB$_{135}$ electrolyte. **a**, C 1s, O 1s, and F 1s XPS spectra of Li metal negative electrodes. **b**, Corresponding atomic ratios at various intervals of Ar^+ sputtering.

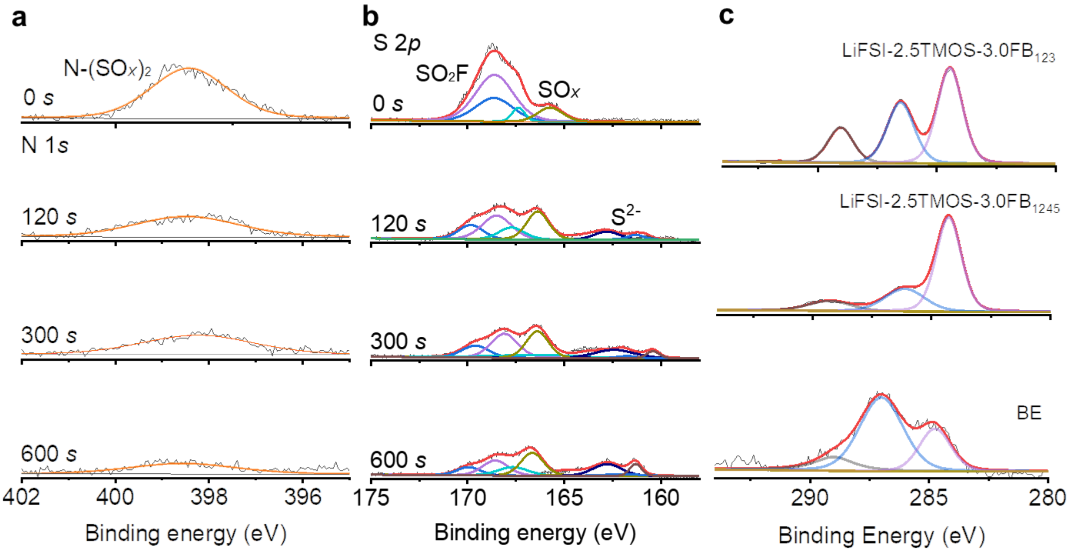

Supplementary Fig. 37 XPS characterization of SEIs formed on cycled Li metal negative electrodes after 10 cycles (0.5 mA cm^2, 4 mAh cm^2) at 25 °C in different electrolytes. a, N 1*s* XPS spectra of Li metal cycled in LiFSI-2.5TMOS-3.0FB$_{135}$ at different etching times (1, 120, 300, 600 s), **b**, S 2*p* XPS spectra of Li metal cycled in LiFSI-2.5TMOS-3.0FB$_{135}$ at different etching times (1, 120, 300, 600 s), **c**, C 1*s* XPS spectra of Li metal cycled in different electrolytes.

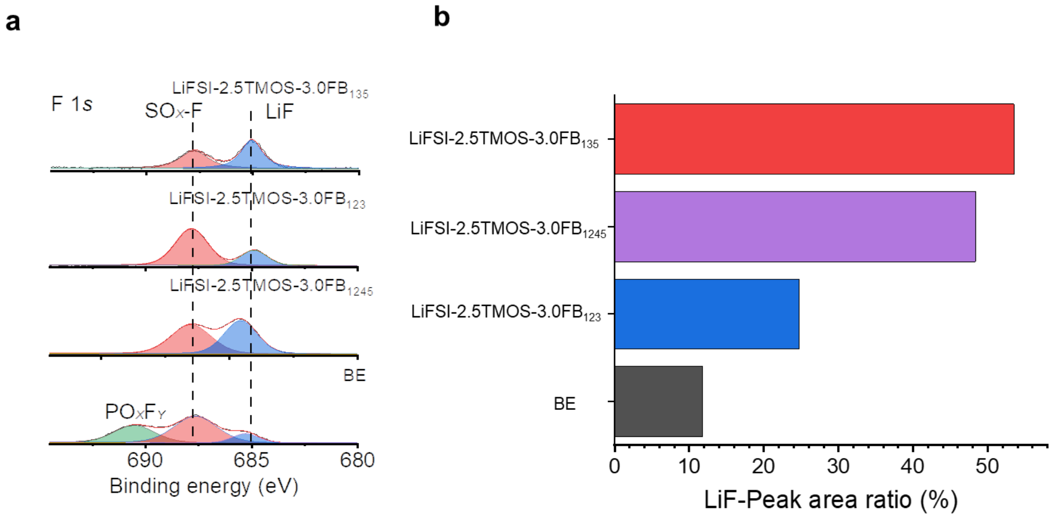

Supplementary Fig. 38 F 1s XPS spectra of Li metal negative electrodes cycled Li metal negative electrodes after 10 cycles (0.5 mA cm^2, 4 mAh cm^2) at 25 ^{o}C in different electrolytes.

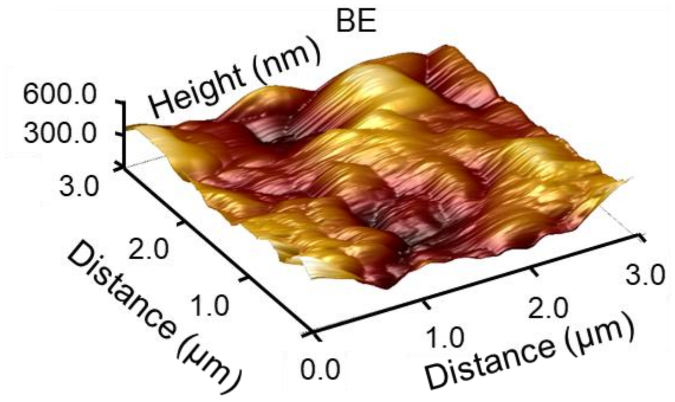

Supplementary Fig. 39 AFM morphology at a region of 3.0×3.0 μm^2 on Li metal negative electrodes from Li||Cu cells after 20 cycles (0.5 mA cm^2, 4 mAh cm^2) at 25 ^{o}C in BE.

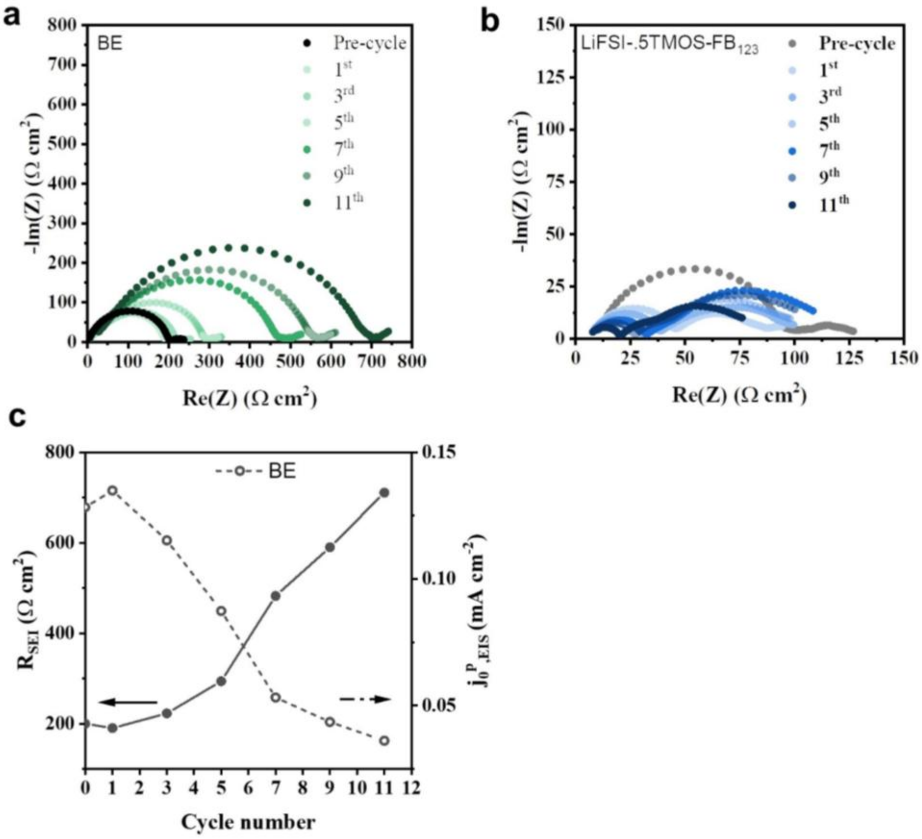

Supplementary Fig. 40 Transport properties and dynamic evolution of SEI in Li||Li cells after different Li plating and stripping cycles (0.5 mA cm^{-2}, 1 mAh cm^{-2}) at 25 ^{o}C in different electrolytes. Nyquist plots of Li/Li cells collected pre- and post-cycling in **a**, BE, **b**, LiFSI-2.5TMOS-FB$_{123}$, **c**, SEI resistance and pseudo-exchange current density for BE.

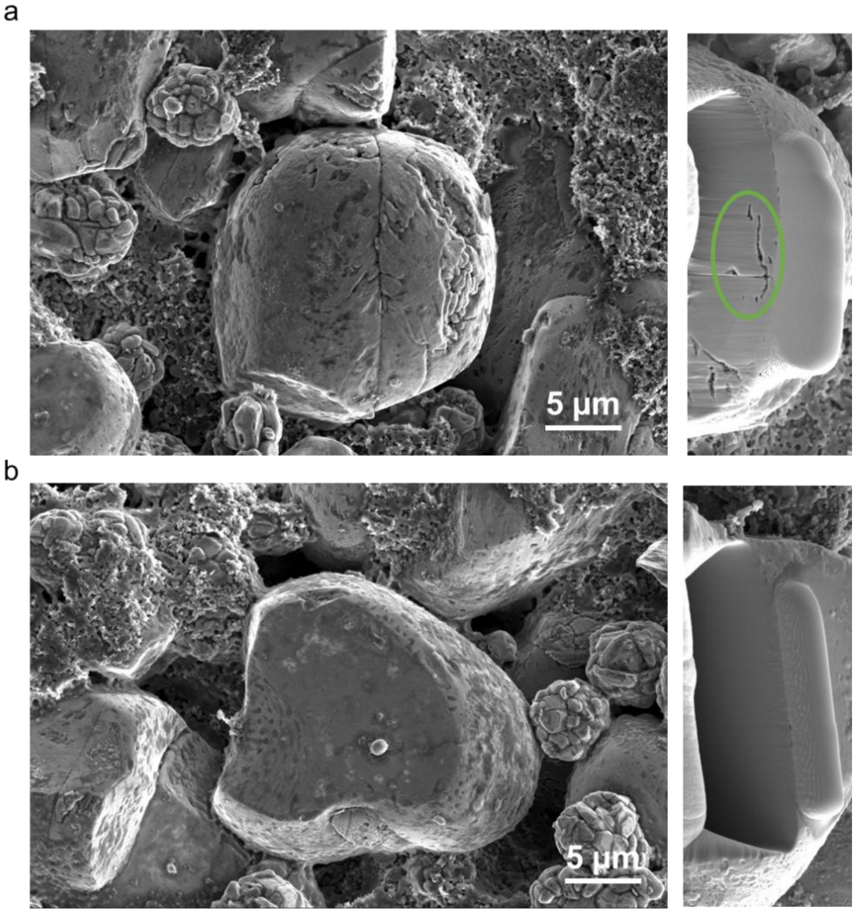

Supplementary Fig. 41 SEM images of LCO in a fully discharged state after 200 cycles (0.2C/0.5C charge/discharge) at 25 °C in different electrolytes. a, LiFSI-2.5TMOS-3.0FB$_{123}$. **b**, LiFSI-2.5TMOS-3.0FB$_{135}$.

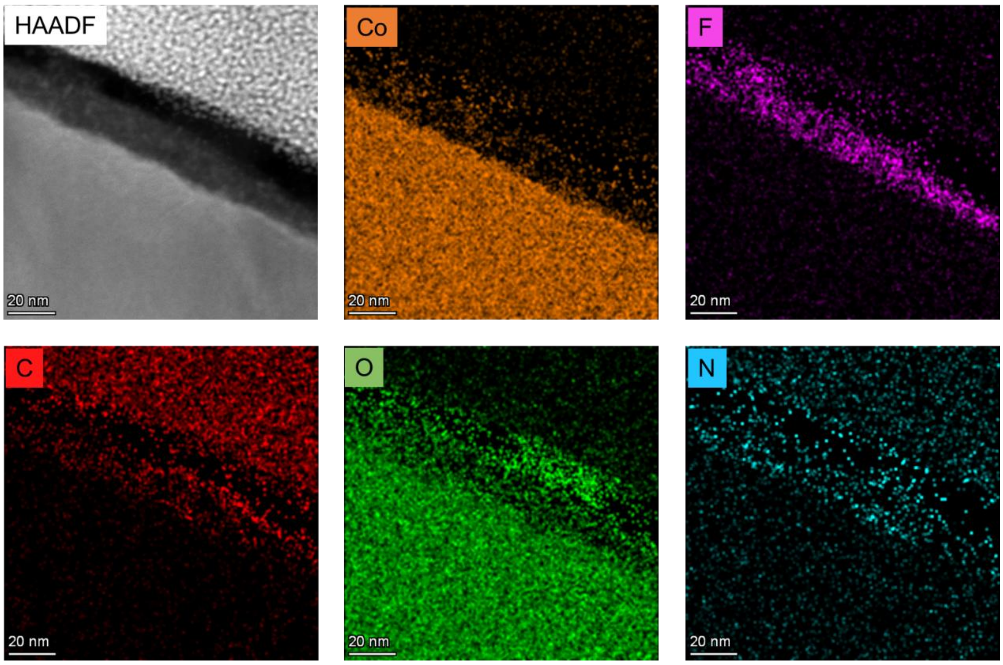

Supplementary Fig. 42 Energy dispersive X-ray analysis (EDX) and corresponding mapping signals of Co, F, C, O and N Characterizations of positive electrode/electrolyte interphase formed in LiFSI-2.5TMOS-3.0FB$_{135}$ electrolyte. LCO in a fully discharged state after 200 cycles (0.2C/0.5C charge/discharge) at 25 °C.

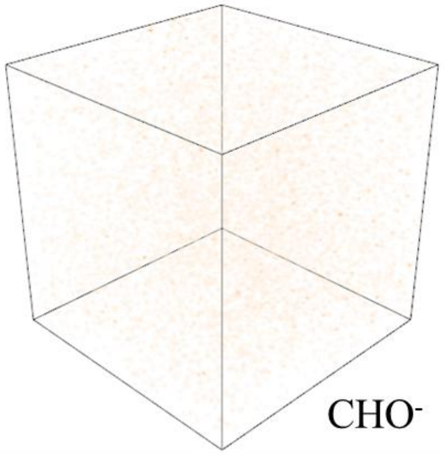

Supplementary Fig. 43 3D maps presenting the distribution of CHO^- of cycled LCO for the cycled LCO positive electrode in a fully discharged state after 200 cycles (0.2C/0.5C charge/discharge) at 25 °C in LiFSI-2.5TMOS-3.0FB_{135}, sample area 50 × 50 µm with 600 s sputter time.

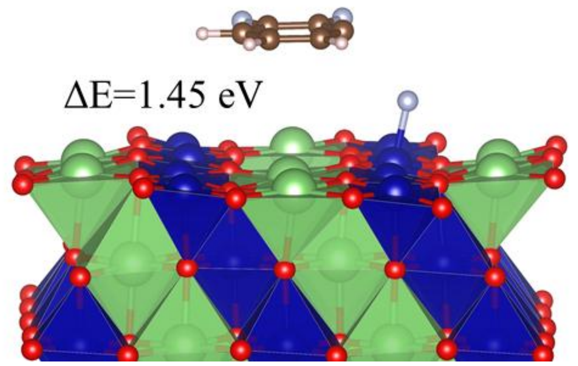

Supplementary Fig. 44 Oxidation reaction energy Δ*E* of F^- transfer for FB_{123} on the delithiated LCO surface. Colour code: green: Li atom, Co: dark blue, red: O atom, cyan: F atom, tan: C atom, white: H atom.

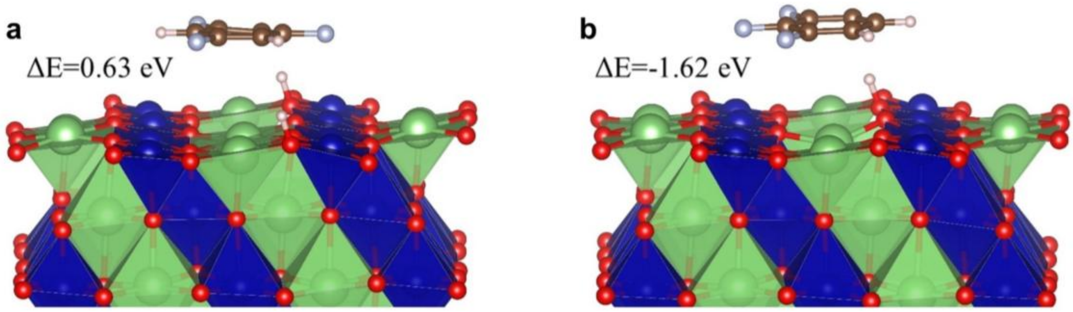

Supplementary Fig. 45 Reaction energies Δ*E* of H-abstraction for different molecules on the delithiated LCO surface. **a**, for FB_{135} molecule. **b**, for FB_{123} molecule. The H-abstraction reaction energies presented in Supplementary Fig. 44 directly reveal the oxidation stability of the electrolyte. Compared with the FB_{135} electrolyte (ΔE = 0.63 eV), the FB_{123} electrolyte spontaneously undergoes H-transfer reactions (-1.62 eV), suggesting the enhanced oxidation stability of LiFSI-2.5TMOS-3.0FB_{135} electrolyte. Colour code: green: Li atom, Co: dark blue, red: O atom, cyan: F atom, tan: C atom, white: H atom.

Supplementary Table 1 The thermodynamic reduction potential of the selected solvent from DFT simulations.

Solvent	Name	Reduction potential (V, *vs.* Li^+/Li)
TEP	Triethyl phosphate	0.73
TMP	Trimethyl phosphate	0.82
TMS	Tetramethylene sulfone	0.95
PC	Propylene carbonate	0.78
DMC	Dimethyl carbonate	0.60
DEC	Diethyl Carbonate	0.89
EA	Ethyl acetate	0.91
MDFA	Methyl difluoroacetate	0.98
DME	1,2-Dimethoxyethane	0.15
1,3-DX	1,3-Dioxane	0.55
1,4-DX	1,4-Dioxane	0.51
THF	Tetrahydrofuran	0.29
Me-THF	2-Methyltetrahydrofuran	0.23
ETH	Ethoxyethane	0.17
TBME	tert-Butyl methyl ether	0.20
TMOS	Trimethyl methoxysilane	0.33
DMMS	Dimethyldimethoxysilicane	0.32
DMOTFS	dimethoxy(methyl)(3,3,3-trifluoropropyl)silane	0.36

Supplementary Table 2 Interaction energies of studied dimer complexes computed at the revDSD-PBEP86-D3(BJ)/jul-cc-pVTZ level (indicated as E_{int}^{DFT}) and SAPT2+/aug-cc-pVDZ (indicated as E_{int}^{SAPT}).

Solvent	Li^{+}-solvent interaction (kcal mol^{-1})			FSI^{-}-solvent interaction (kcal mol^{-1})		
	G_{bind}^{DFT}	E_{int}^{DFT}	E_{int}^{SAPT}	G_{bind}^{DFT}	E_{int}^{DFT}	E_{int}^{SAPT}
TMP	-64.40	-74.66	-73.40	-6.77	-10.98	-11.76
TEP	-60.44	-70.95	-72.05	-9.02	-10.81	-11.40
TMS	-64.03	-74.93	-75.66	-4.15	-8.22	-8.37
PC	-65.45	-75.69	-76.60	-13.88	-17.23	-17.76
DMC	-42.98	-53.44	-53.33	-3.38	-7.56	-7.79
DEC	-43.52	-54.52	-55.65	-2.65	-6.82	-6.61
EA	-57.05	-68.02	-68.99	-4.15	-8.22	-7.47
FEA	-54.38	-65.36	-64.99	-3.92	-8.01	-7.24
DME	-55.76	-66.21	-66.45	-2.87	-6.98	-6.937
1,3-DX	-36.71	-47.35	-46.89	-6.63	-10.52	-11.06
1,4-DX	-37.62	-48.32	-47.74	-7.18	-11.03	-10.51
THF	-44.77	-55.62	-53.20	-3.19	-7.23	-6.94
Me-THF	-39.55	-50.01	-51.69	-3.49	-7.56	-7.28
ETH	-34.88	-45.63	-46.69	-2.45	-6.36	-6.44
TBME	-29.99	-40.58	-42.5	-2.15	-6.46	-5.33
TMOS	-37.38	-47.96	-47.49	-1.44	-5.76	-5.54
DMOTFS	-35.46	-46.02	-44.86	-2.24	-6.34	-6.24
DMMS	-34.98	-45.62	-43.62	-1.85	-5.99	-5.81
CFH	-11.03	-22.08	-22.83	-5.42	-9.36	-8.87
NME	-11.52	-22.31	-23.09	-7.12	-10.95	-10.23
PMP	-9.76	-20.57	-21.37	-5.01	-9.03	-8.35
FB_{135}	-13.88	-24.62	-24.45	-2.49	-6.82	-6.72
FB_{123}	-6.81	-17.56	-17.13	-3.48	-7.62	-6.99
FB_{124}	-5.72	-16.37	-16.65	-3.18	-7.23	-7.12

FB_{1245}	-6.32	-16.99	-17.99	-3.39	-7.52	-7.02
FB	-7.57	-18.32	-17.71	-3.38	-7.52	-7.28
2FB	-7.13	-17.69	-17.59	-3.47	-7.55	-7.37
TTE	-7.11	-17.66	-17.30	-3.25	-7.62	-9.04
BTFE	-6.27	-16.82	-17.03	-7.11	-10.36	-9.63
TFEO	-6.31	-16.86	-17.54	-5.39	-9.35	-10.22

Supplementary Table 3 Physical components of the interaction energies for Li^+-solvent dimer complexes calculated by using SAPT method.

Solvent	E_{ele} (kcal mol^{-1})	E_{exc} (kcal mol^{-1})	E_{ind} (kcal mol^{-1})	E_{dis} (kcal mol^{-1})
TMP	-62.32	24.96	-34.13	-1.91
TEP	-60.86	24.36	-33.69	-1.87
TMS	-65.02	25.88	-35.26	-1.27
PC	-63.57	22.68	-33.68	-2.04
DMC	-47.49	25.36	-30.41	-0.79
DEC	-46.89	24.86	-32.57	-1.05
EA	-55.62	22.37	-34.37	-1.37
FEA	-51.68	21.70	-33.74	-1.28
DME	-57.21	23.97	-31.82	-1.39
1,3-DX	-40.37	23.11	-28.68	-0.97
1,4-DX	-41.59	22.97	-28.11	-1.00
THF	-43.98	22.68	-30.67	-1.23
Me-THF	-44.27	23.62	-29.68	-1.36
ETH	-38.69	21.69	-28.64	-1.05
TBME	-35.96	22.09	-26.94	-1.69
TMOS	-40.54	19.26	-25.79	-0.42
DMOTFS	-36.65	20.36	-26.21	-2.36
DMMS	-36.69	21.36	-25.61	-2.68
CFH	-17.56	14.63	-17.92	-1.98
NME	-17.51	14.52	-18.35	-1.76
PMP	-16.97	14.73	-17.04	-2.09
FB_{135}	-16.91	13.32	-17.99	-2.87
FB_{123}	-18.06	19.98	-17.13	-1.92
FB_{124}	-17.89	19.66	-16.64	-1.78
FB_{1245}	-17.92	19.11	-16.38	-2.80
FB	-18.41	19.95	-17.71	-1.54

2FB	-17.79	19.36	-17.59	-1.57
TTE	-17.51	20.26	-17.31	-2.74
BTFE	-16.99	20.36	-17.02	-3.38
TFEO	-17.05	19.98	-17.53	-2.94

Supplementary Table 4 Physical components of the interaction energies for FSI^--solvent dimer complexes calculated by using SAPT method.

Solvent	E_{ele} (kcal mol^{-1})	E_{exc} (kcal mol^{-1})	E_{ind} (kcal mol^{-1})	E_{dis} (kcal mol^{-1})
TMP	-33.24	57.31	-16.58	-19.25
TEP	-32.47	58.7	-17.27	-20.36
TMS	-29.69	55.98	-16.98	-17.69
PC	-32.09	53.86	-17.69	-21.84
DMC	-29.17	60.32	-17.57	-21.37
DEC	-28.67	59.88	-17.08	-20.741
EA	-27.82	57.36	-15.68	-21.33
FEA	-25.70	58.91	-15.77	-24.69
DME	-26.17	55.213	-15.59	-20.39
1,3-DX	-25.63	53.68	-16.75	-22.36
1,4-DX	-25.67	53.72	-16.87	-21.69
THF	-24.36	54.66	-12.66	-24.58
Me-THF	-24.68	55.66	-13.25	-25.01
ETH	-23.25	50.36	-14.33	-19.22
TBME	-20.67	53.36	-15.69	-22.33
TMOS	-12.13	27.37	-4.85	-15.93
DMOTFS	-16.32	28.63	-4.90	-13.65
DMMS	-14.95	26.82	-5.32	-12.36
CFH	-19.52	36.56	-5.23	-20.68
NME	-19.86	36.65	-5.65	-21.35
PMP	-18.68	36.69	-5.72	-20.65
FB_{135}	-18.35	35.77	-3.57	-20.57
FB_{123}	-21.03	32.67	-4.35	-14.28
FB_{124}	-20.89	33.57	-4.29	-15.51
FB_{1245}	-19.94	33.21	-4.68	-15.61
FB	-21.37	31.56	-5.01	-12.46

2FB	-22.45	32.78	-4.87	-12.83
TTE	-23.36	36.60	-5.69	-16.59
BTFE	-21.96	34.30	-4.99	-16.98
TFEO	-22.37	34.61	-5.23	-17.23

Supplementary Table 5 Li metal CE in Li||Cu cells using different combinations of solvent pairs.

Type	Electrolytes	Mole ratio (LiFSI:1.0)	CE (%)[a]	Test method
Type I + Type II	TMP-TTE	1.0:1.25:1.5	98.4	Aurbach
	TMP-FB_{123}	1.0:1.25:1.5	99	Aurbach
	DMC-FB_{123}	1.0:1.25:1.5	99.1	Aurbach
	DME-FB_{123}	1.0:1.25:1.5	99.2	Aurbach
	DMC-TTE	1.0:1.25:1.5	99.3	Aurbach
	DME-TTE	1.0:1.25:1.5	99.3	Aurbach
	TMS-BTFE	1.0:1.25:1.5	98.4	Aurbach
	TMS-FB_{123}	1.0:1.25:1.5	98.8	Aurbach
Type I + Type IV	TMOS-FB_{1245}	1.0:2.5:3.0	99.3	Aurbach
	TMOS-FB_{124}	1.0:2.5:3.0	99.3	Aurbach
	MeTHF-FB_{123}	1.0:2.5:3.0	99.1	Aurbach
	TBME-FB_{123}	1.0:2.5:3.0	99.2	Aurbach
	TMOS-TTE	1.0:2.5:3.0	99.3	Aurbach
	TBME-BTFE	1.0:2.5:3.0	99.3	Aurbach
Type III + Type IV	MeTHF-FB_{135}	1.0:2.5:3.0	99.65	Aurbach
	TBME-FB_{135}	1.0:2.5:3.0	99.6	Aurbach
	TMOS-FB_{135}	1.0:2.5:3.0	99.7	Aurbach
	TMOS-CFH	1.0:2.5:3.0	99.55	Aurbach
	TBME-MNE	1.0:2.5:3.0	99.57	Aurbach
	MeTHF-CFH	1.0:2.5:3.0	99.6	Aurbach

[a] Three cells were measured for calculating CE of each electrolyte.

Supplementary Table 6 MD benchmark of pure solvent used in this work.

Simulated solvent	Force field	Charge model	Numbers of solvents	Simulation cell size (Å)	Density (g cm^{-3})	Dielectric constants
TMOS	Charge-scaled classical	1.14*CM1A	160	32.86	0.78	3.36
		1.2*CM1.5		33.01	0.77	3.56
		RESP		33.45	0.74	3.42
	Polarizable	--		33.16	0.76(0.756)	3.25
DME	Charge-scaled classical	1.14*CM1A	160	30.21	0.87	7.42
		1.2*CM1.5		30.31	0.86	7.34
		RESP		30.67	0.83	7.16
	Polarizable	--		30.43	0.85(0.867)	7.20
FB_{135}	Charge-scaled classical	1.14*CM1A	160	29.91	1.31	8.10
		1.2*CM1.5		30.14	1.27	7.63
		RESP		30.40	1.25	7.42
	Polarizable	--		30.19	1.27(1.28)	7.50
FB_{123}	Charge-scaled classical	1.14*CM1A	160	30.01	1.30	8.63
		1.2*CM1.5		30.15	1.28	7.92
		RESP		30.24	1.27	7.36
	Polarizable	--		30.21	1.26(1.28)	7.86

Note: red text is the experimental value.

Supplementary Table 7 Calculated structures and physical parameters for various electrolytes.

Simulated electrolyte		LiFSI-2.5TMOS-3.0FB$_{135}$					
Force field		Charge-scaled classical				Polarizable	Quantum-based
Scaling factor		0.65	0.70	0.75	0.80	--	--
Numbers of species		80 LiFSI, 174 TMOS, and 241 FB$_{135}$					5 LiFSI, 11 TMOS, and 15 FB$_{135}$
Simulation cell size (Å)		47.76	46.54	45.99	45.18	46.21	18.21
Density (g cm^{-3})		0.99	1.07	1.11	1.17	1.09	1.12 (Exp.)
Coordination number (cutoff: 2.8 Å)	Li-O(FSI)	2.51	2.73	2.84	2.95	2.89	2.82
	Li-O(TMOS)	1.37	1.28	1.21	1.13	1.19	1.25
	Li-F(FB$_{135}$)	0.20	0.14	0.12	0.10	0.11	0.06
Simulated electrolyte		LiFSI-2.5TMOS-3.0FB$_{123}$					
Force field		Charge-scaled classical				Polarizable	Quantum-based
Scaling factor		0.65	0.70	0.75	0.80	--	--
Numbers of species		80 LiFSI, 174 TMOS, and 241 FB$_{123}$					5 LiFSI, 11 TMOS, and 15 FB$_{123}$
Simulation cell size (Å)		46.83	45.84	45.17	44.55	44.92	17.95
Density (g cm^{-3})		1.05	1.12	1.17	1.22	1.19	1.17 (Exp.)
Coordination number (cutoff: 2.8 Å)	Li-O(FSI)	2.31	2.49	2.72	2.88	2.75	2.78
	Li-O(TMOS)	1.45	1.36	1.29	1.2	1.33	1.29
	Li-F(FB$_{123}$)	0.25	0.18	0.15	0.13	0.13	0.08
Simulated electrolyte		LiFSI-2.5DME-3.0FB$_{123}$					
Force field		Charge-scaled classical				Polarizable	Quantum-based
Scaling factor		0.60	0.65	0.70	0.75	--	--
Numbers of species		80 LiFSI, 174 DME, and 241 FB$_{123}$					5 LiFSI, 11 DME, and 15 FB$_{123}$
Simulation cell size (Å)		44.72	44.10	43.74	43.28	43.62	17.41
Density (g cm^{-3})		1.16	1.21	1.24	1.28	1.25	1.23 (Exp.)
Coordination number (cutoff: 2.8 Å)	Li-O(FSI)	0.92	1.16	1.23	1.34	1.19	1.21
	Li-O(DME)	3.77	3.67	3.42	3.32	3.48	3.50
	Li-F(FB$_{123}$)	0.03	0.02	0.02	0.01	0.01	0.05

Supplementary Table 8 Structure parameters for MD simulations starting from different initial configurations.

Simulated electrolyte	Numbers of solvents	Initial packing protocol	Initial Simulation cell size (Å)	Final Simulation cell size (Å)
LiFSI-2.5DME-3.0FB_{135}	80 Li, 80 FSI, 174 DME, 241 FB_{135}	Separate	38.00*38.00*76.00	35.15*35.15*69.42
		Uniform		35.28*35.28*69.53
LiFSI-2.5TMOS-3.0FB_{135}	80 Li, 80 FSI, 174 TMOS, 241 FB_{135}	Separate	38.00*38.00*81.00	35.26*35.26*74.29
		Uniform		35.41*35.41*74.52

Supplementary Table 9 EIS fitting results of cell cycled in different electrolytes.

Electrolyte	Cycling number	R_b (ohm)	R_s (ohm)	R_{ct} (ohm)	χ^2
LiFSI-2.5DME-3.0FB_{135}	50	29.4±0.5	45.6±0.7	54.3±0.5	1.36
	300	32.6±0.3	113.0±1.3	60.7±0.9	1.52
LiFSI-2.5DME-3.0FB_{123}	50	30.4±0.2	63.5±0.7	78.4±1.5	1.32
	300	50.1±0.4	150.1±1.6	160.2±2.1	1.99

Supplementary references:

1. Emamian, S., Lu, T., Kruse, H. & Emamian, H. Exploring Nature and Predicting Strength of Hydrogen Bonds: A Correlation Analysis Between Atoms-in-Molecules Descriptors, Binding Energies, and Energy Components of Symmetry-Adapted Perturbation Theory. **40**, 2868-2881 (2019).
2. Řezáč, J., Riley, K. E. & Hobza, P. S66: A well-balanced database of benchmark interaction energies relevant to biomolecular structures. *J. Chem. Theory Comput.* **7**, 2427-2438 (2011).
