# Supplementary material for: A path towards high lithium-metal electrode coulombic efficiency based on electrolyte interaction motif descriptor
Source: Nat Commun. 2025 May 20;16:4672. doi: 10.1038/s41467-025-59955-0 (PMC12092653; doi:10.1038/s41467-025-59955-0)
Supplement: Supplementary file 2 — Description of Additional Supplementary Files [file 41467_2025_59955_MOESM2_ESM.pdf]

Description of Additional Supplementary Files

File Name: Supplementary Data 1
Description: Optimized structures of calculated projected density of states of different electrolytes in figure 3c and Supplementary Fig. 18.

File Name: Supplementary Data 2

Description: Initial and final structures to calculate F- and H-transfer reaction of different molecules on the delithiated $LiCoO_2$ surface in figure 6c and Supplementary Fig. 44 and 45.
